# Coexistence of Fish Species in a Large Lowland River: Food Niche Partitioning between Small-Sized Percids, Cyprinids and Sticklebacks in Submersed Macrophytes

**DOI:** 10.1371/journal.pone.0109927

**Published:** 2014-11-03

**Authors:** Małgorzata Dukowska, Maria Grzybkowska

**Affiliations:** Department of Ecology and Vertebrate Zoology, Faculty of Biology and Environmental Protection, University of Łódź, Łódź, Poland; University of Yamanashi, Japan

## Abstract

In the spring and summer of each year, large patches of submersed aquatic macrophytes overgrow the bottom of the alluvial Warta River downstream of a large dam reservoir owing to water management practices. Environmental variables, macroinvertebrates (zoobenthos and epiphytic fauna, zooplankton) and fish abundance and biomass were assessed at this biologically productive habitat to learn intraseasonal dynamics of food types, and their occurrence in the gut contents of small-sized roach, dace, perch, ruffe and three-spined stickleback. Gut fullness coefficient, niche breadth and niche overlap indicated how the fishes coexist in the macrophytes. Chironomidae dominated in the diet of the percids. However, ruffe consumed mostly benthic chironomids, while perch epiphytic chironomids and zooplankton. The diet of dace resembled that in fast flowing water although this rheophilic species occurred at unusual density there. The generalist roach displayed the lowest gut fullness coefficient values and widest niche breadth; consequently, intraspecific rather than interspecific competition decided the fate of roach. Three-spined stickleback differed from the other fishes by consuming epiphytic simuliids and fish eggs. The diet overlap between fishes reaching higher gut fullness coefficient values was rather low when the food associated with the submersed aquatic macrophytes was most abundant; this is congruent with the niche overlap hypothesis that maximal tolerable niche overlap can be higher in less intensely competitive conditions.

## Introduction

Dams usually affect downstream characteristics, like flow regimes, river-channel geomorphology, water and sediment quality, aquatic environment and biota [1]–[3]. The effects of the dam related river management practice may be evident for many kilometers of downstream reaches; one such effect is the development of submersed aquatic macrophytes (SAM) [4], [5]. The main factors and processes controlling macrophyte status in lowland rivers are discharge and/or current velocity, light, substrate and nutrients, while the role of the first two is of most fundamental importance, as these hydrological parameters control instream macrophyte colonization, establishment and persistence [6]. The presence of SAM may be considered as a very important component of riverine biota, causing an increase in habitat structural complexity in alluvial lotic ecosystems. The water plants serve as a substrate for epiphyton, constituting a rich foraging habitat for macroinvertebrates [7], shelter against predation, heterogeneous substrate for co-existence and, to a small extent, a direct, food source [8]–[10]. Besides, the submersed plants' morphological characteristics (size, number and orientation of leaves and steams) influence both invertebrate [8], [10] and fish distribution [11]–[13]. Thus SAM may support a high density of small fish individuals, because submersed plant beds offer protection from predators by hindering predators' foraging activities [14]. The foraging activity of vertebrate predators may decline monotonically with increasing habitat complexity [15].

At the river bed macrophyte patches, fish may exploit prey types from three ecological formations: zooplankton (especially numerous below dam reservoirs), fauna dwelling on the surface of vegetation (epiphytic fauna, mainly several taxa of Chironomidae and Simuliidae) and benthos. Submersed plants create favourable conditions for pelophilous forms like most of Oligochaeta and Chironomidae by extensive particle trapping and accumulation of a fine-grained, nutrient enriching sediment [16]–[18]. Thus the development of SAM on the alluvial bed river attracts many small fish individuals. Being a little competitive, the coexistence of these species is possible if there are differences in their responses to limiting resources. The species-specific differences that allow such coexistence can be considered as species' niche with four major axes: resources, predators, space and time [19], [20].

Untypical, but abundant development of SAM has been observed in the large lowland alluvial Warta River downstream of the Jeziorsko Reservoir every year as an effect of a low discharge in late spring and summer [10]. Every early autumn large volumes of water start to be released through the reservoir dam sluices, and the SAM habitat is torn out of the bottom or gets inundated with the bottom substrate [3], [10]. The trophic relationships among this rich but temporary habitat have been investigated with regard to the primary and secondary invertebrate consumers (gathering collectors, scrapers and predators) and the tertiary consumer of three-spined stickleback (*Gasterosteus aculeatus* L.), as well as percids [7], [18], [21], [22].

For a long time every year, however, the Jeziorsko tailwater has been dominated by cyprinids [3], [22]–[25], many small individuals of which were also foraging in the tailwater's SAM. The trophic impact of the fishes on this habitat has not been investigated, although we have long expected that all types of the rich food resources connected with the SAM (zoobenthos, epiphytic fauna and zooplankton) are exploited by these (and other) fishes. Consequently, the main objective of the present study was to identify patterns in the feeding of the five predominant fish species occurring in that area (roach, dace, perch, ruffe and three-spined stickleback), in order to evaluate their trophic niches' breadths and overlaps in relation to resources, time and space. To this end, we investigated in detail the gut contents and intraseasonal changes in the diet of these five fish species there.

## Study area

**Figure 1:**
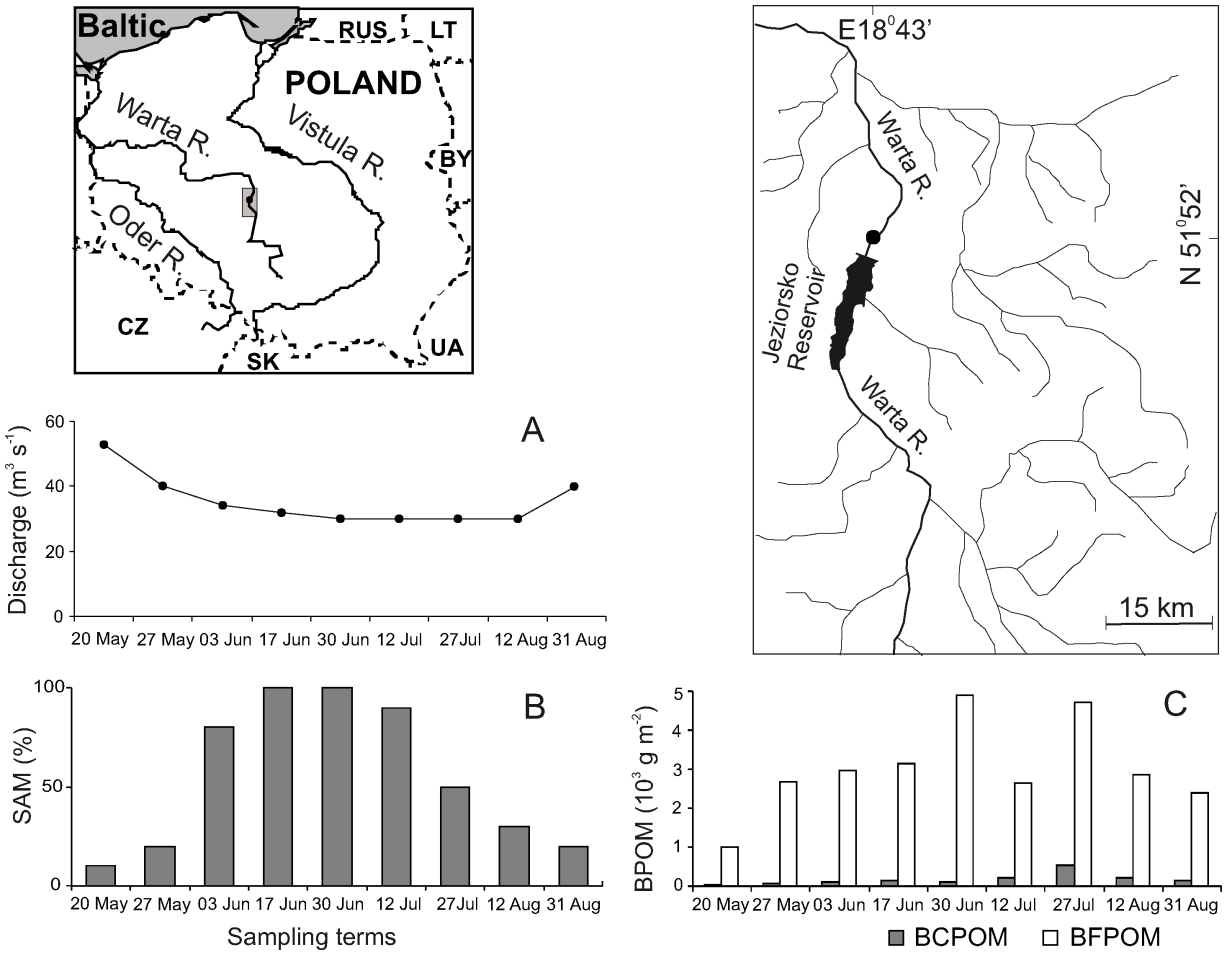
Study area with the marked sampling site in the tailwater of the Jeziorsko Reservoir on the Warta River. Parameters of the site over the study period: A. discharge; B. SAM -% of river bottom covered by hydrophytes; C. BFPOM and BCPOM, two fractions of benthic particulate organic matter (details in the text).

The Warta River rises 380 meters above sea level, is 808 km long and empties into the Oder River at 13 meters above sea level. Its catchment area is ca. 53 710 km^2^ and its slope ranges from 2.0–1.0‰ in the upper course, and from 0.3–0.1‰ in the middle and lower courses [26]. The study site was established in this lowland alluvial river about 1.5 km downstream of the dam of the large Jeziorsko Reservoir, whose maximal surface area is 42.3 km^2^ (Fig. 1). At the investigated site, the Warta River is about 60 m wide, with a maximum depth of 2.5 m in the erosion zone.

During sampling in 2004, similarly as in a few former years, the discharge of the Warta River below the dam was quite different in comparison with the natural, upstream reach, especially in summer, when its flow stabilized at a much lower level than in other seasons. One consequence of this phenomenon was the appearance of submersed macrophytes, which started to spread each year from the summer of 1992 along a short stretch of the tailwater [3], [27]. Detailed site descriptions can be found in Grzybkowska et al. [10], [28].

## Material and Methods

The sampling was conducted in 2004, 18 years after the reservoir started functioning routinely and 10 years after the construction of a hydroelectric plant. In the investigated reach, large patches of *Potamogeton pectinatus* L. and small patches of *Potamogeton lucens* L. gradually developed since late spring through late summer in the transitional riverbed zone, which is located between the depositional zone, close to a bank, and the midriver channel (Fig. 1). The percentage of river bottom covered by macrophytes, samples of particulate organic matter and inorganic substrate, macrophytes, zoobenthos and epiphytic fauna, zooplankton, and fish, were collected, at the same time, within an area sized 40 m by 2.5 m, and extending along the bank and along the transitional zone. The area was randomly selected within the zone. The samples were obtained twice a month from May through late August. As a result, nine samples of each biotic and abiotic component were collected.

On each sampling occasion each sample of benthos (containing benthic invertebrates, particulate organic and inorganic matter) consisted of five subsamples (each subsample covered 100 cm^2^ of stream-bed area collected with a tabular sampler of a catching area of 10 cm^2^). The sampling places were uniformly distributed within the sampled area. The invertebrates of the samples were sorted from the detritus and benthic sediments by hand and preserved in 10% formalin. All invertebrates from these quantitative samples were counted and their wet weight (w.w.) assessed; these data were used to estimate the biomass of zoobenthos. Most invertebrates were classified to the lowest taxonomic level of the dominant macrobenthic group, while chironomids were identified to the species level when possible. As the exact identification on the basis of their larvae was often impossible, we reared their immature stages in the laboratory from additional qualitative samples taken each time in order to obtain larval and pupal skins, and imagines.

These samples were also used to determine the organic matter content in the bottom sediment. For this purpose a 1 mm mesh sieve was used to separate benthic particulate organic matter (BPOM) into:>1 mm (coarse – BCPOM) and <1 mm (fine – BFPOM) [29]. Next, the benthic organic matter was dried at 60°C for two days, weighed, ashed at 600°C for two hours and reweighed. A more detailed description of these methods can be found in Grzybkowska et al. [10], Grzybkowska & Dukowska [27].

To estimate the amount of dry weight of plants growing in the study site, a special frame (0.5×0.7 m) was placed on the riverine bottom and all the *Potamogeton* within the frame was collected. This procedure was repeated three times on each sampling occasion. In the laboratory, the pondweeds were dried for 24 hours at 65°C to estimate their dry weight (d.w.).

Five subsamples of the epiphytic fauna settled on *Potamogeton* were collected on each sampling occasion. Each of the subsamples consisted of three fragments of stems (about 20 cm long, on average) of the plants cut off under the water surface, stored in plastic containers, and preserved in 10% formalin in the fields. In the laboratory, the plant material was removed from the containers and the invertebrates were washed off the plants, sorted by hand, identified to the species level when possible, counted, had their wet weight assessed. The obtained data were recalculated to estimate the biomass of epiphytic invertebrates per 1 m^2^ of *Potamogeton* covering the riverine bottom on given sampling occasions.

To evaluate the density of zooplankton (mainly Cladocera), 0.03 m^3^ samples of river water were filtered through a planktonic net, of 50 µm mesh size, and preserved in formalin with riverine water in the fields. In the laboratory, individuals were identified to the species level and counted. The biomass of the zooplankton was estimated on the basis of suitable equations [30].

To assess fish density and gut contents, fish were caught in the sampled area using an electric current of 220 V and 3 A supplied from a backpack battery generator. A single pass CPUE sampling was carried out, which consisted in one person wading against the water current with an anode dipnet and another one with a bucket for collecting stunned fish along the longitudinal axis of the sampled area. The sampling period was 15 minutes on each occasion. As the equipment was battery-powered fish were not scared by noise, hence no barrier preventing fish from escaping was necessary. Immediately after the capture, fish were anaesthetised (MS-222, tricaine methanesulfonate) and then preserved in 4% formalin. The field studies did not involve endangered or protected species. The Polish Angling Association in Konin (a tenant of the water body, director Jerzy Olejnik, 1 Wyspiańskiego Str., 62–510 Konin) issued the permit to conduct the field study (see Fig. 1 for details of the study area location). Electrofishing was performed with the license No 1180/01 for the operation of electric fishing tools, and the other procedures were conducted under the permission No 219/2011 to perform experiments on animals, issued by the University of Lodz, and the individual licence No 6/2006 to perform experiments on animals according to the Law on the protection of animals and the recommendations of the ICLAS.

In the laboratory, the total length (TL) of each analysed fish specimen was measured to the nearest 1 mm, and weighed to the nearest 0.1 g. The gut contents of all investigated individuals (n = 242) were analysed using a stereomicroscope and microscope. Prey types from the whole gut length, after identification to the lowest possible taxonomic category, were counted and weighed (w.w.), except zooplankton, the biomass of which was estimated in the same way as described above.

The gut fullness coefficient (FC) was calculated by the formula [31]:
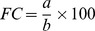



where:

*a* – total gut content weight (g)

*b* – weight of fish (g)

Niche breadth (B_n_) was calculated using Levins' index:
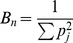
where:

*B_n_ –* Levins' measure of niche breadth

*p_j_* – proportion of food type *j*

*n* – number of possible food types.

We standardized the trophic niche values (ranging from 0 to 1) using the Hurlbert formula [32]:
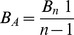
where:

*B_A_* – Levins' standardized niche breadth

*B_n_ –* Levins' measure of niche breadth

*n* – number of possible food types.

The interspecific diet overlap among the investigated species was calculated using the Schoener overlap index [33]:
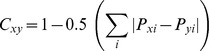
where:

*C*_xy_ – is the overlap index ranging from 0 (no overlap) to 1 (complete overlap)

*P_xi_* – is the proportion of food type *i* of species *x*

*P_yi_* – is the proportion of food type *i* of species *y*

It is worth noting that C*_xy_*>0.6 is considered biologically significant [34].

The cluster analysis was also applied for identifying fish species' groups with similar gut contents.

The Kruskal-Wallis test and the post-hoc Dunn test in the STATISTICA software package, v. 10 [35], [36] were applied to compare the percentage of the most frequent food items in the diet, the gut fullness coefficient, as well as diet niche breadth, to determine whether significant changes during the investigated period occurred. The significance level of the tests was α = 0.05.

## Results

### Intraseasonal dynamics of environmental parameters of the SAM habitat

The development of macrophytes started in late May. It gradually intensified the cumulation of benthic fine particulate organic matter on the bottom, which strengthened the possibilities of the development of pelophilous zoobenthos. The highest biomass values of *P. pectinatus* (over 210 g d.w. m^−2^) and the highest density of BFPOM were recorded during the second half of June 2004; at that time the macrophytes were also covered with filaments of green algae *Cladophora glomerata* (L.) Kutz. The intraseasonal dynamics of the above characteristics are presented in Fig. 1. Inorganic substratum was mostly fine and coarse sand, as well as some gravel.

### Fish assemblage attributes

**Figure 2:**
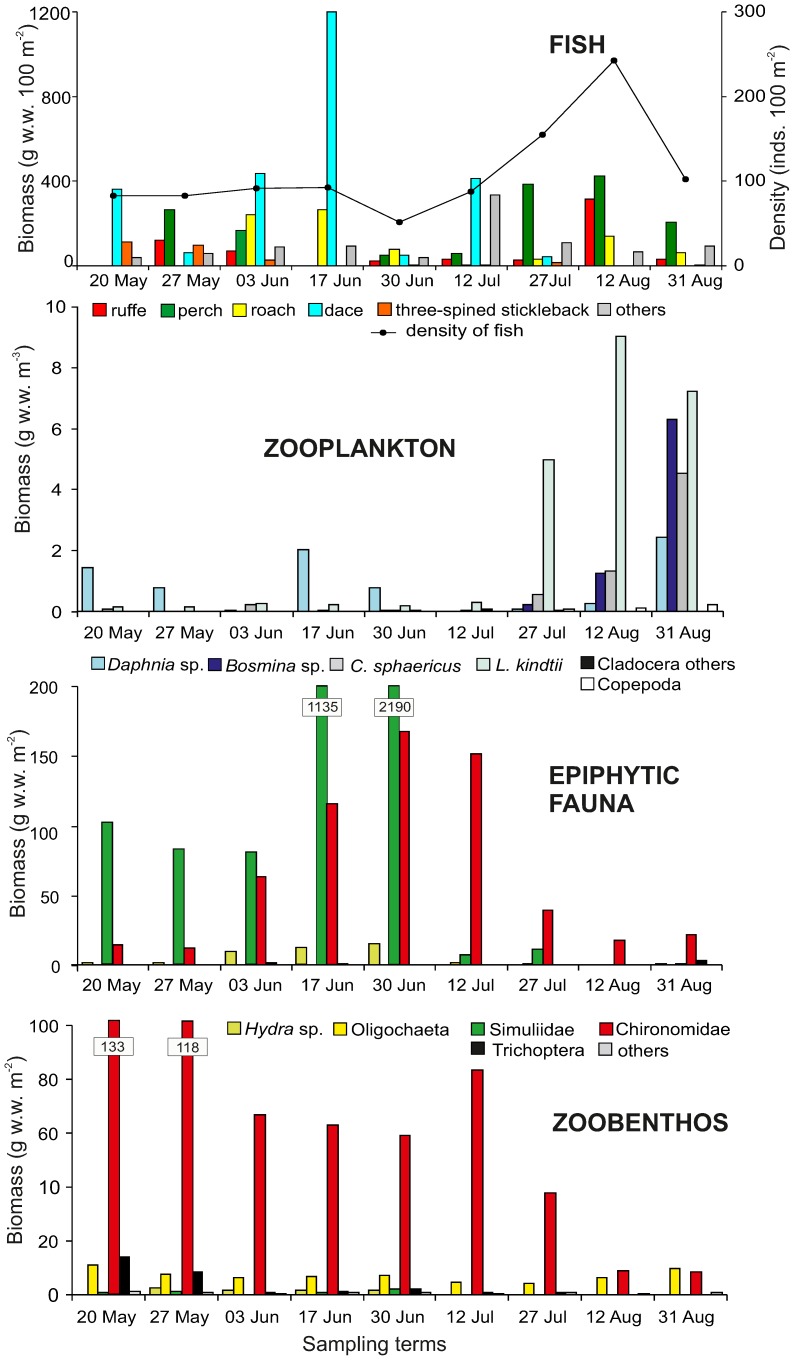
Fish and their food resources. Intraseasonal dynamics of biomass and density of fish and of their food resources (zooplankton, epiphytic fauna and zoobenthos) over the study period.

Over the investigated period the total of 14 fish species inhabiting *Potamogeton* patches in the SAM habitat were captured. The dominant species was perch (*Perca fluviatilis* L.), constituting 32.7% of the total fish density and 23.0% of their total biomass. In order of their decreasing importance in total fish biomass, the following species were: dace (*Leuciscus leuciscus* (L.) and roach (*Rutilus rutilus* (L.)), 38.2% and 12.1% of the biomass and 15.5% and 18.2% of the density, respectively. In turn ruffe (*Gymnocephalus cernuus* (L.)), accounted for 9.1% of the biomass and 11.8% of the density, while three-spined stickleback (*Gasterosteus aculeatus* L.) reached 4.0% of the biomass and 11.8% of the density. Spined loach (*Cobitis taenia* L.), gudgeon (*Gobio gobio* (L.) and ide (*Leuciscus idus* (L.), were captured less frequently, constituting with other fish species 13.6% of the biomass and 10.0% of the density (Fig. 2).

All guts of the analysed individuals of the five species were filled to various extent (n = 242). The characteristics of the fish are presented in Table 1.

**Table 1:** Characteristics of analysed fish specimens in the SAM habitat of the Warta River, Poland.

Fish species	N		B (g)	TL (mm)	FC
*Gymnocephalus cernuus* (L.) (ruffe)	41	mean R	2.7 0.8–16.0	63 44–113	2.4 0.6–7.3
*Perca fluviatilis* L. (perch)	55	mean R	3.3 0.8–14.2	66 41–102	0.8 0.1–4.9
*Rutilus rutilus* (L.) (roach)	76	mean R	1.8 0.13–44.2	56 24–147	0.4 0.03–1.5
*Leuciscus leuciscus* (L.) (dace)	37	mean R	16.6 10.8–29.3	116 100–136	1.0 0.1–2.1
*Gasterosteus aculeatus* L. (three-spined stickleback)	33	mean R	2.4 0.3–4.2	54 31–70	4.1 0.5–7.2

N – number of specimens, B – biomass, TL – total length, FC – gut fullness coefficient, R – range of values.

### Food resources associated with SAM

#### Microcrustaceans

Over the whole investigated period Cladocera dominated in water column, reaching 99% of the zooplankton biomass of all investigated periods. Copepoda were very scarce, with the maximum in late August. The maximum values of cladoceran biomass were recorded in August due to the presence of large-sized predator *Leptodora kindtii* (Focke) (49.7% of the total cladoceran biomass during the study period). The most numerous specimens in water column were the small sized species of *Chydorus sphaericus* (O. F. Müller) (which constituted only 15.1% of zooplankton biomass over the investigated period), *Bosmina* spp. (17.1%) and large size taxa, *Daphnia* spp. (17.2%) (Fig. 2).

#### Epiphytic and benthic fauna connected with *Potamogeton*

Simuliids dominated among the macrophytes, amounting to 84.7% of the biomass of the total epiphytic fauna, but were abundant only till late June. Different seasonal dynamics was showed by chironomids (14.1% of total epiphytic fauna biomass), which reached the highest biomass in June and July but occurred throughout the study period. Main chironomid species, on *Potamogeton*, were: *Cricotopus sylvestris* (Fabricius) (Orthocladiinae), and *Parachironomus gracilior* (Kieffer) (Chironominae-Chironomini). Other epiphytic invertebrates (including *Hydra* sp.) occurred rarely (1.2%) (Fig. 2).

Chironomidae dominated in the benthos over the whole investigated period, reaching 83.7% of total benthic invertebrate biomass, and their abundance decreased over time. The dominant chironomids in benthos were represented by *Chironomus riparius* Meigen, *Dicrotendipes nervosus* (Staeger), *Glyptotendipes cauliginellus* (Kieffer) and *Polypedilum* spp. (Chironominae-Chironomini), as well as *Cricotopus bicinctus* (Meigen) (Orthocladiinae) and *Paratanytarsus* (Tanytarsini). Oligochaeta (9.3%) were present at the same level over the whole studied period, while Trichoptera (4.1%, mainly *Hydropsyche* spp.) were present mostly in May. The macrophyte bed was also inhabited by *Hydra* sp. (1.2%), especially in May and June (Fig. 2).

### Trophic attributes of fish

**Figure 3:**
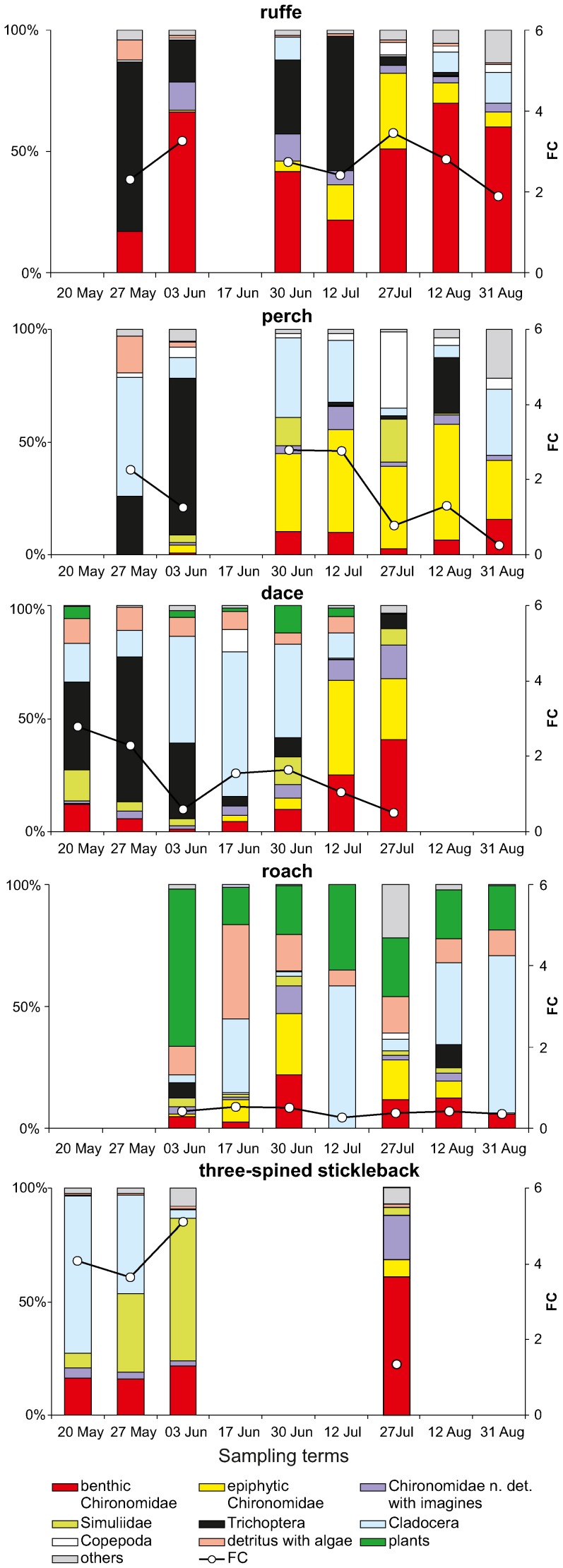
Fish diet composition. Food items and gut fullness coefficient (FC) of each fish species over the study period.

Fish associated with the SAM exploited five main food categories: zooplankton (mainly Cladocera), benthos (Chironomidae during the whole studied period, and Trichoptera at the beginning of the investigated period), epiphyton (Chironomidae and Simuliidae), plants, detritus with algae, including the filamentous ones (Fig. 3).

In dace, Cladocera occurred on six of the seven sampling occasions on which the fish species was recorded. However, the biomass of Cladocera first rapidly increased to mid June, when they became dominant, and then decreased to none in late July. Both epiphytic and benthic chironomids became the first/second most important food category of dace in July. It is worth noting that dace also exploited imagines from the water surface. Three-spined stickleback consumed mainly Cladocera in May, which were replaced by Simuliidae in June. Benthic Chironomidae were the second/third most important food category in May and June but almost completely dominated the diet of this species in July. In ruffe, benthic chironomids were the main food category consumed on all sampling occasions, except late May and early July, when Trichoptera dominated instead. Epiphytic chironomids appeared in ruffe's diet since late June. Cladocerans and epiphytic chironomids dominated the diet of perch to a similar extent on most of the sampling occasions, although copepods became a subdominant and trichopterans a decisive dominant on one occasion each. In turn in roach, plants were the dominant food component on the first and a subdominant on all other sampling occasions. Cladocera were the main food type on three sampling occasions, in July and August. Detritus and algae were present during the whole study period (Fig. 3).

From the point of view of given food categories, in the course of the whole investigated period, the main component of roach diet were plants (about 28.2% of gut contents), while for other species it was the accessory food (Kruskal-Wallis test, H = 178.49, p<0.0001), among which higher percentages of SAM (about 4%) were recorded for dace.

Chironomidae were consumed by all fish during the whole studied period; these dipterans, living in the bottom, were the main food of ruffe and statistically more important than those of other fish species (Kruskal-Wallis test, H = 108.51, p<0.0001). In turn, epiphytic chironomid taxa were consumed by perch more than by other fishes (Kruskal-Wallis test, H = 57.87, p<0.0001). Other dipterans, Simuliidae, were the main food category (about 26.8% of gut contents) only for three-spined stickleback (Kruskal-Wallis test, H = 33.92, p<0.0001), and to a lesser extent, for dace and perch (about 6% of gut contents). Trichoptera (large size larvae represented by *Hydropsyche*) were exploited to a greater extent by dace, ruffe and perch, than by roach and three-spined stickleback (Kruskal-Wallis test, H = 61.16, p<0.0001); perch consumed *Hydropsyche* larvae at a lower level than dace (post-hoc Dunn test, p<0.036). Detritus with algae was the main component in the gut contents of roach and dace and statistically different than of the other studied fishes (Kruskal-Wallis test, H = 142.24, p<0.0001). Cladocerans were exploited by all fish; the lowest biomass of this prey was recorded in ruffe guts (Kruskal-Wallis test, H = 28.03, p<0.0001). Other microcrustaceans, Copepoda, were an important diet component for perch only (Kruskal-Wallis test, H = 60.08, p<0.0001). Complementary food types (included in the “others” category) were ephemeropterans, other insects and *Asellus aquaticus* (Isopoda), as well as ostracods.

Over the investigated season, the fullness coefficients of these five analysed fish species were statistically different (Kruskal-Wallis test, H = 130.58, p<0.0001). As showed by the post-hoc Dunn test the differences did not exist only between ruffe and three-spined stickleback and between perch and dace. Moreover, for the two last species the highest variability in FC values was noted: for perch from 0.25 at the end of August to 2.8 at the end of June, while for dace from 0.5 at the end of July to 2.8 at the end of May. However, the lowest variability was observed for roach: from 0.3 at the end of August to 0.6 in the middle of June. For the whole studied period, the highest FC values were noted for three-spined stickleback (at the beginning of June) (Fig. 3).

**Figure 4:**
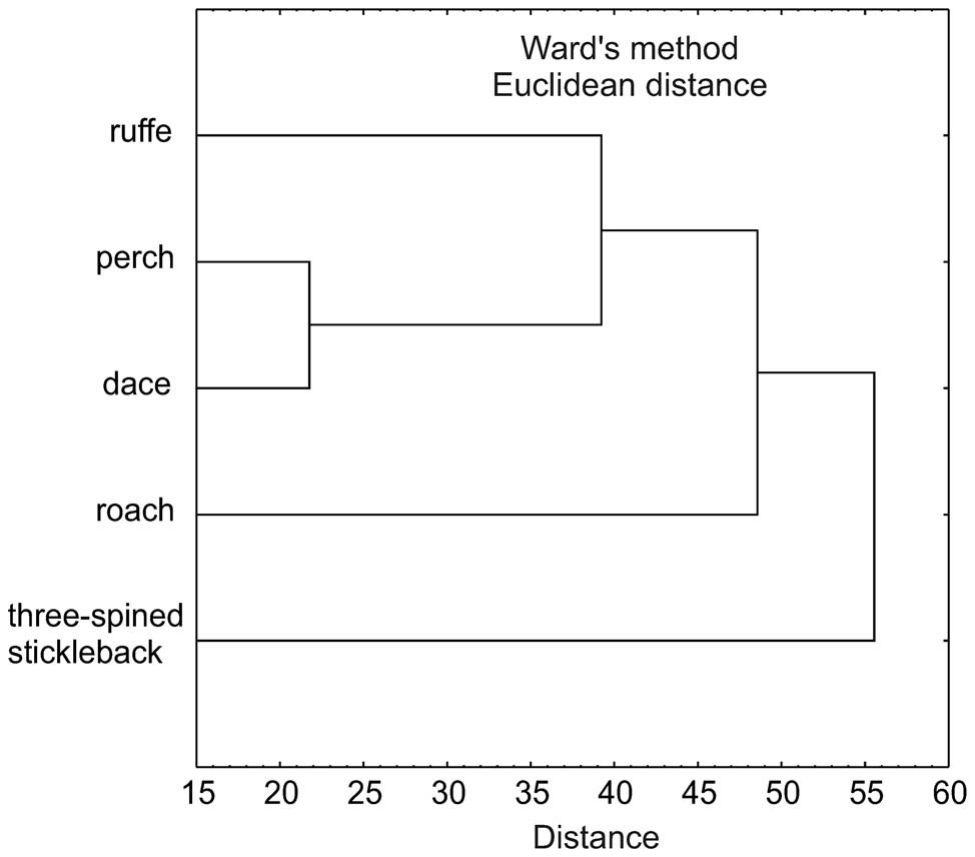
Similarity of fish species in terms of diet. Dendrogram resulting from the cluster analysis performed on gut contents of each species over the whole investigated period.

The diet compositions of the five fish species were analyzed using hierarchical cluster analysis, which distinguished three feeding groups: 1) three-spined stickleback which consumed epiphytic simuliids (main prey), and occasionally fish eggs, 2) roach, feeding mainly on plant materials, detritus and algae, zooplankton, and Chironomidae as an important complementary food category, 3) dace, perch and ruffe, which ate mainly animal materials (aquatic and terrestrial invertebrates) (Fig. 4).

**Figure 5:**
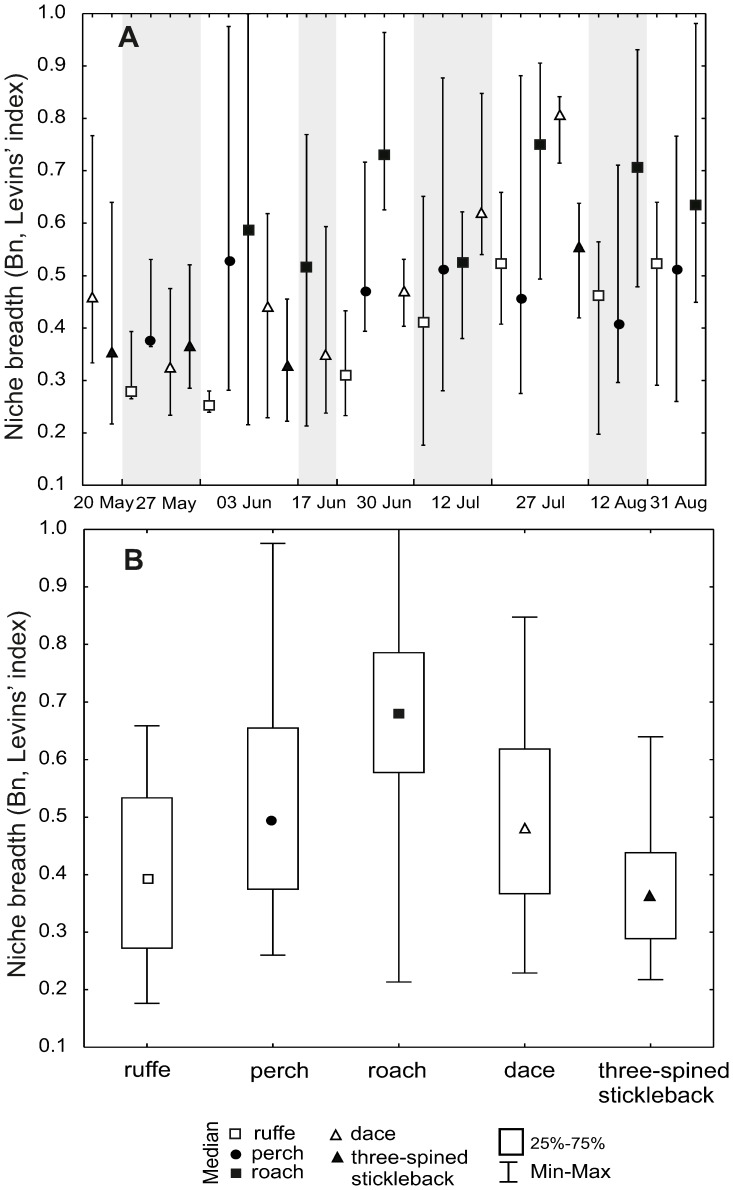
Food niche breadth of fishes. A. Intraseasonal variation in the niche breadth (medians of Levins' index, B_n_) of each species. B. Median of the niche breadth for each species over the whole studied period. Vertical bars represent the interquartile range.

Niche breadths (Levins' index) of these species were statistically different (Kruskal-Wallis test, H = 125.02, p<0.0001) and varied seasonally. On each sampling occasion when roach and ruffe occurred, the niche breadth of the former was always much wider than of the latter (Fig. 5A). The evaluation of niche breadth revealed a less diverse diet of typical benthic fishes, such as ruffe, while the most diverse diet was in the case of roach. As regards niche breadths, three non-hierarchical groups of species (Kruskal-Wallis test, post hoc Dunn test, H = 77.015, p<0.0001) were distinguished: the first group was roach, the second group were dace and perch, and the third were ruffe and three-spined stickleback (Fig. 5A and B).

**Figure 6:**
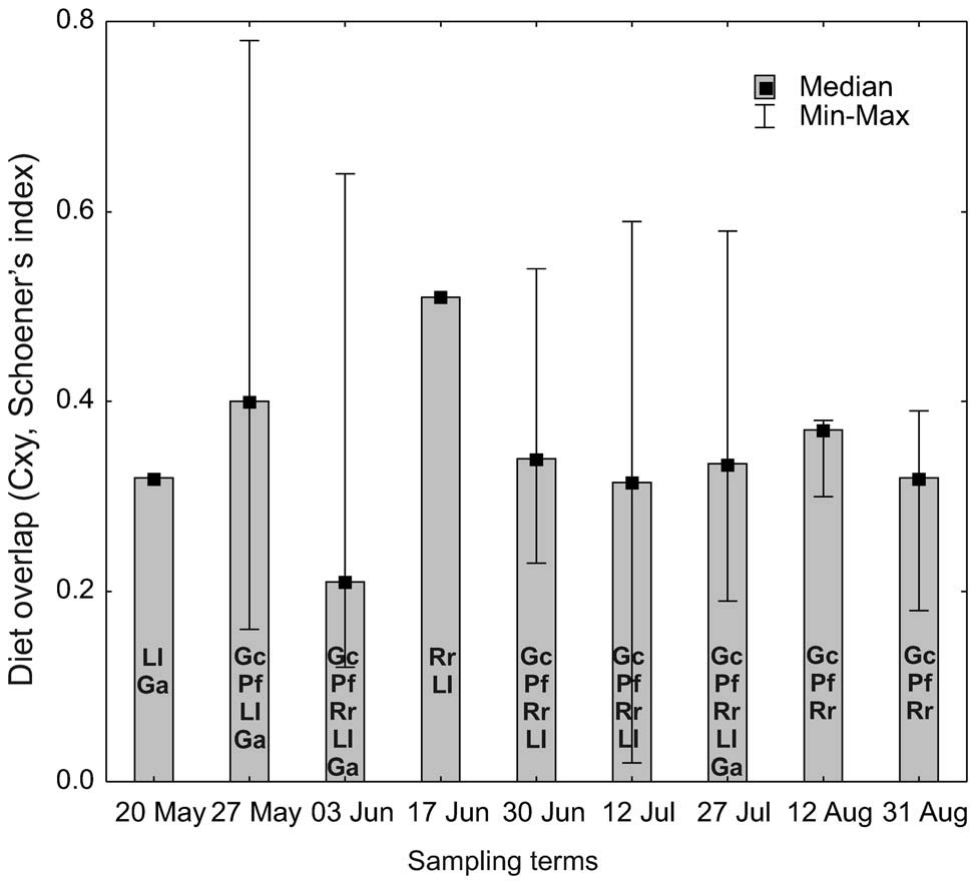
Interspecific diet overlaps. Interseasonal variation in the diet overlap (medians of the Schoener index, C_xy_) between perch (Pf), ruffe (Gc), roach (Rr), dace (Ll) and three-spined stickleback (Ga); the above abbreviations in parentheses come from scientific names of fish species. Other explanations as in Fig. 5.

The Schoener interspecific diet overlap index did not differ between cyprinids, percids and three-spined stickleback over the studied period. However, this index varied seasonally, reaching the highest value between ruffe and dace (0.78 at the end of May when both species exerted pressure on the *Hydropsyche*) while the lowest ones were attained a few times: between roach and other fish (4 times lower or equal to 0.2) and between three-spined stickleback and other species (also 4 times lower, Fig. 6).

## Discussion

### Submersed aquatic macrophytes in large rivers

In the two recent decades, the bed zone between the bank and midriver channel of the tailwater stretch of the Warta River has undergone a seasonal regime shift from an alluvial to a macrophyte-dominated system. Such processes also took place in many other impacted large rivers. As this phenomenon is usually believed to exert negative environmental impacts, attempts to counteract it, using various methods, particularly artificial flooding, were applied in these rivers [37]. SAMs are rather undesirable elements in large rivers because they modify the in-river environment mainly by altering river flows, and by decreasing the depth of trapping sediments, which is also beneficial for large populations of pelophilous macrobenthic fauna. However, SAMs are the excellent surface for epiphyton. Some groups of invertebrates, represented by grazers, like snails, macrocrustaceans and cladoceran zooplankters, are able to protect aquatic macrophytes by removing epiphytes and phytoplanktonic algae. The plants themselves help the process of defense because they are a source of biochemical compounds that, on one hand, negatively affect the growth of algae (allelopathy), but, on the other hand, may attract grazers. Many fish species occupying this rich habitat also positively affect SAMs as main predators foraging on epiphytic invertebrates. Consequently, these vertebrates are an important part of a complex network of relations between nutrients, phytoplankton, epiphytes, herbivorous invertebrates and benthos [38]–[40].

### Fish foraging among vascular plants

From the beginning of the functioning of the Jeziorsko Reservoir (1986) in the Warta River downstream from its dam the diversity of fish declined regularly, while the number of these vertebrates showed quite opposite trends, mainly due to perch and roach, and to a lesser degree, to ruffe. This process of fish decline concerns, for example, dace, a rheophilic species (i.e. riverine specialist), which was present sporadically, and even absent in some years [3], .

Dace, which exploits open water with fast water currents, usually suffer from human-induced changes in aquatic environment and is thus considered a good bioindicator [41]–[43]. The over-representation of dace among dense leaves and stems of *Potamogeton* recorded in the impacted stretch of the Warta River was rather unexpected. Dace showed a very high plasticity to food resources connected with SAM, feeding on larger, easily catchable benthic organisms, epiphytic fauna and/or zooplankton as well as imagines from water surface (mainly Chironomidae) which died after mating and/or rested on the surface above its pupal exuviae after eclosion. However, dace reached the highest value of FC when consuming large *Hydropsyche* larvae and at that time their niche overlapped that of ruffe. In June the diet of dace comprised also Cladocera, and the FC of dace was reduced. The presence of dace in SAM may testify to the greater attractiveness of this temporary habitat than the sandy mid-channel of the alluvial Warta River, where only tiny organisms, such as some Oligochaeta and Chironomidae, occasionally occur. Also, the potential migration of dace towards the depositional, SAM-less zone (see Material and methods), would not bring many benefits to the fish because the mud covering that zone is frequently flooded, or, to a high extent, exposed (to air) due to pulse water releases from the reservoir. Therefore, at the depositional zone benthic fauna was remarkably impoverished as compared with the natural river section (upstream of the reservoir); virtually no insects (except some families of Diptera), Hirudinea and Oligochaeta, were collected there [3], [27], [28], [44]. Summing up, dace food spectrum in the very biologically productive SAM habitat was similar to those in other ecosystems [45]–[47] in which dace may also display daily migrations to and from feeding places [48]. It is worth noting that this species also shows ontogenetic shifts in its food spectrum [49].

Three-spined stickleback was the second species for which over-representation in SAM as compared with its abundance in the whole tailwater reach was observed [50], but only at the beginning of the studied period. According to Bańbura [50], three-spined stickleback feed mainly on Copepoda, Cladocera, larvae of Chironomidae, and seasonally Mollusca, Oligochaeta and fish eggs, but none of these items exceeded 25% of the gut contents. This finding is not in accordance with the present observations. In the macrophyte beds of the Warta River, the food of this species varied intraseasonally; the extraordinary development of the epiphytic fauna caused a foraging shift from Cladocera (constituting at the beginning of the study period about 70% of gut contents, and consisting mainly of large sized species of *Daphnia*, of reservoir origin, [51]) to epiphytic fauna, especially to the largest (oldest) larvae of the dominant dipterans, both simuliids and chironomids (the majority of gut contents in June). Low niche overlap between three-spined stickleback and other species was the effect of consuming epiphytic simuliids as the main prey and other prey types, such as fish eggs (the highest values of FC occurred when the species fed on these elements), rather untypical food items for other fish. This finding is confirmed by a dendrogram resulting from the cluster analysis.

The choice of older (larger) dipteran individuals by stickleback, as stated in our earlier paper [22], is consistent with the optimal foraging theory [52] - organisms forage in such a way as to maximize their net energy intake per unit time.

Ruffe, as a typical benthivorous fish, and partially three-spined stickleback and dace, play an important role in the internal nutrient cycling; firstly, during the searching for food (through bioturbation), secondly, during the translocation of nutrients from the bottom to the water column realized via their digesting and egestion. Ruffe, a small but aggressive fish native to Europe and Asia, may be classified rather among specialists [18], [53]–[56] than generalists [57]. In lakes, ruffe may efficiently forage in deeper and darker bed patches due to its very sensitive lateral line system and the light-reflecting *tapetum lucidum* in its eye [58], [59]. In the Warta River, this vertical gradient of food selection by ruffe was confirmed by Dukowska et al. [18], while its horizontal movement for searching food is rather unprofitable, similar to dace [3], [27], [28]. Overlaps between ruffe diet and those of other species were at the same level (from 0.21 to 0.34) regardless of ruffe's preference for benthic chironomids and *Hydropsyche*.

According to some studies, ruffe do not undergo ontogenetic dietary shifts, and remain the bottom feeders throughout the life cycle [57].

In rivers, high diversity and abundance values of fish are usually strongly associated with a high degree of trophic specialization (low amplitude of individual trophic niches) and a small degree of overlap between the diets of species constituting an assemblage [60]. But in the impacted stretch of the Warta River fish diversity has been low from the beginning of the functioning of the Jeziorsko Reservoir, although the abundance of several species was very high [24], [25]. Each year, when the amount of water released from the reservoir increases, a mass escapement of fish of eurytopic species, predominantly juvenile stages, gets intensified. This mainly concerns the species that have attained reproductive success in the reservoir in a given year (including mostly perch and roach, but also ruffe) and may lead to an increase in the abundance of these species (two or three orders of magnitude higher than in the pre-impoundment period or in the upstream river stretch) [3], [24], [61].

Perch, a potential competitor for ruffe [56], [62], is included among generalists. It shows an ontogenetic shift in its diet during its life history, from zooplankton (at the onset of exogenous feeding) to macroinvertebrates, and from macroinvertebrates to fish [23], [63]–[67]. However, the adults of this species may also be classified as trophic generalists switching frequently between piscivorous, zooplanktivorous, and benthivorous feeding style depending on the food resources in the environment. In the Warta River, in spite of this mode of foraging in areas drastically overcrowded by perch [61], the species starved also at the end of the present study period, despite the increased transport of zooplankton from the reservoir [18], [51]. This is in accordance with assumptions of the theory of the ideal free distribution stating that an increased number of individuals in a given patch of resources reduces its quality, through either increased scramble competition or increased interference competition (including aggressive behavior) [68], [69].

In the Warta River, diet overlap between perch and ruffe was rather low (the Schoener index, range 0.21–0.34) as perch preferred to eat epiphytic chironomids, simuliids and *Daphnia*, while ruffe chose large sized benthic chironomids and trichopterans (if they were present) and occasionally zooplankton [18]. It is worth stressing that the identification of prey, mainly Chironomidae, of these two fish species, to genus (mainly) or species level helped to determine the food resource partition between these two closely related species, because these fishes generally consumed different chironomid taxa [18].

In the Warta River, the second numerically most abundant species connected with SAM was roach. The interaction between perch and roach has been studied by many ecologists, especially in lentic ecosystems [67], [70], [71]. Both young roach, which fed mainly on detritus, chironomids and zooplankton, as well as older individuals, which consumed mostly detritus, filamentous algae, vascular plants, macroinvertebrates and molluscs, represented typically omnivorous feeding habitats, and are thus considered generalists [71]–[73]. Besides, roach may be classified into opportunists. If huge amounts of easily accessible food appear, roach use it immediately; for example, during chironomid emergences the alimentary tracts of roach are filled in with numerous pupae of these insects [74]. For each chironomid pupa being in a water column is a very dangerous experience (as the pupa may then end up as the prey of macroinvertebrates and/or fish), however, at that time zooplankton, mainly *Daphnia*, is released from fish predation [75].

In the present material, an insufficient amount of food available for roach (starvation?) is indicated by the low values of its FC. Its niche breadth was the largest one of those of all the five species during the whole studied period. The niche overlap between this species and other fishes was low, thus we may suppose that rather an intraspecific than interspecific competition decided the fate of small roach in this overcrowded environment. Even if certain individuals of this eurytopic species die because of competitive interactions, they will soon be replaced by juveniles migrating massively from the reservoir [25], [61], [76]. It is worth mentioning that the consumption of such food categories as algae and detritus may have resulted in a slow growth rate [73]. A large spectrum of food types consumed by roach, larger than that of perch, was recorded by other ecologists [67], [70].

## Conclusions

In the SAM habitat the diet overlap was highest when the SAM patches, and the food resources associated with them, were most developed (twice during the investigated period). For the first time at the beginning of SAM development, when large-sized *Hydropsyche* were the main food category for ruffe, perch and dace, and for the second time throughout June and the first half of July, when benthic and/or epiphytic Chironomidae were the basic food items for these species. This finding is congruent with the niche overlap hypothesis saying that maximal tolerable niche overlap can be higher in less intensely competitive situations, i.e. in environments with lower demand/supply ratios [77].

One of the most important attributes of organisms to avoid direct overlap in the use of resources is diversification of body size of individuals of given species [60]. We put focus on three (time, space and resource) of the four niche axes of each of the five fish species living among the submersed macrophytes and we concluded that their diets only partly overlap, which allows them to coexist in this temporary, very rich habitat.

## Supporting Information

Table S1 **Food items (% of biomass) in alimentary tracts of ruffe (Gc), perch (Pf), dace (Ll), roach (Rr) and three-spined stickleback (Ga).**
(PDF)
