# Supplementary material for: Coexistence of Fish Species in a Large Lowland River: Food Niche Partitioning between Small-Sized Percids, Cyprinids and Sticklebacks in Submersed Macrophytes
Source: PLoS One. 2014 Nov 3;9(11):e109927. doi: 10.1371/journal.pone.0109927 (PMC4217722; doi:10.1371/journal.pone.0109927)
Supplement: Table S1 — Food items (% of biomass) in alimentary tracts of ruffe (Gc), perch (Pf), dace (Ll), roach (Rr) and three-spined stickleback (Ga). (PDF) [file pone.0109927.s001.pdf]

Table S1. Food items (% of biomass) in alimentary tracts of ruffe (Gc), perch (Pf), dace (Ll), roach (Rr) and three-spined stickleback (Ga).

fish specie	sampling t	Chironomidae imagines	Chironomus riparius	Dicrotendipes sp.	Glyptotendipes cauliginellus	Polypedilum sp.	Parachironomus gracilior	Tanytarsus sp.	Paratanytarsus sp.	Cladotanytarsus mancus	Cricotopus bicinctus	Cricotopus sylvestris	Rheocricotopus sp.	Chironomidae others	Simuliidae	Trichoptera	Daphnia sp.	Bosmina sp.	Leptodora kindtii	Cladocera others	Copepoda	Others	detritus with algae	plants	sand	Total
Gc	27 May	0.000	0.000	0.000	4.669	0.973	0.000	0.000	0.000	0.000	8.755	0.000	0.000	0.000	0.000	71.984	0.389	0.000	0.000	0.000	0.000	3.891	9.339	0.000	0.000	100
Gc	27 May	0.000	0.000	0.000	7.042	0.000	0.000	0.000	0.000	0.000	11.737	0.000	0.000	0.000	0.000	70.423	0.469	0.000	0.000	0.469	0.000	2.347	7.512	0.000	0.000	100
Gc	27 May	0.000	0.000	0.000	6.849	0.000	0.000	0.000	0.000	0.000	17.123	0.000	0.000	0.000	0.000	61.644	0.000	0.000	0.000	0.685	0.000	6.849	6.849	0.000	0.000	100
Gc	03 Jun	0.000	0.000	0.000	5.224	1.493	0.000	0.000	0.000	0.000	34.515	0.000	0.000	3.545	0.000	52.239	0.093	0.000	0.000	0.093	0.000	0.000	0.933	0.000	1.866	100
Gc	03 Jun	0.342	0.000	0.000	0.000	0.000	0.000	0.000	0.000	0.000	81.608	0.000	0.000	14.782	0.000	0.000	0.941	0.000	0.000	0.000	0.000	0.000	0.958	0.000	1.369	100
Gc	03 Jun	2.458	0.000	0.000	0.000	0.000	0.000	0.000	0.000	0.000	61.455	0.000	3.933	15.241	0.000	6.883	0.983	0.000	0.000	0.123	0.123	5.900	0.934	0.000	1.967	100
Gc	03 Jun	0.000	0.000	0.000	0.000	0.000	0.000	0.000	0.000	2.090	64.437	0.000	0.000	14.110	0.000	16.723	0.000	0.000	1.045	0.000	0.131	0.000	0.941	0.000	0.523	100
Gc	03 Jun	1.017	8.138	0.000	7.630	0.000	0.000	0.000	0.000	0.000	66.124	0.000	0.000	8.342	0.000	8.138	0.254	0.000	0.000	0.000	0.000	0.000	0.102	0.000	0.254	100
Gc	30 Jun	0.412	4.940	3.294	4.940	1.235	0.000	0.000	1.647	0.000	24.393	2.882	0.000	22.478	0.000	8.234	19.555	0.000	0.000	0.103	0.000	4.117	0.947	0.000	0.823	100
Gc	30 Jun	0.000	2.243	4.487	0.000	0.000	0.000	0.000	2.243	0.000	61.918	6.057	0.000	19.686	0.000	0.000	0.841	0.000	0.000	0.280	0.000	1.122	0.953	0.000	0.168	100
Gc	30 Jun	0.414	1.656	1.656	4.967	0.000	0.000	0.000	0.000	0.000	30.132	2.649	0.000	15.563	0.000	4.139	34.975	0.000	1.242	0.000	0.414	0.414	0.952	0.000	0.828	100
Gc	30 Jun	0.000	0.000	0.000	0.000	0.000	0.000	0.000	0.000	0.000	18.809	0.000	0.000	0.000	0.000	78.370	0.000	0.000	1.450	0.000	0.000	0.000	0.980	0.000	0.392	100
Gc	30 Jun	0.000	6.955	8.560	0.000	0.000	0.000	0.000	1.070	0.000	41.460	2.996	0.000	10.673	0.000	25.679	0.000	0.000	0.401	0.067	0.000	0.535	1.070	0.000	0.535	100
Gc	30 Jun	0.000	4.164	4.164	0.000	0.000	4.164	0.000	0.000	0.000	18.633	2.602	3.643	3.296	0.000	53.782	1.388	0.000	0.000	0.000	0.000	3.470	0.694	0.000	0.000	100
Gc	12 Jul	2.955	4.298	9.401	0.000	1.612	4.835	1.343	1.343	0.000	3.223	2.417	4.835	1.880	0.000	59.898	0.000	0.000	0.000	0.081	0.000	0.806	1.074	0.000	0.000	100
Gc	12 Jul	13.245	22.075	8.830	0.000	4.415	8.830	2.208	2.208	0.000	0.000	6.623	6.623	0.000	0.000	17.660	0.000	0.000	0.000	0.000	0.000	5.298	1.987	0.000	0.000	100
Gc	12 Jul	2.955	4.298	9.401	0.000	1.612	4.835	1.343	1.343	0.000	3.223	2.417	4.835	1.880	0.000	59.898	0.000	0.000	0.000	0.081	0.000	0.806	1.074	0.000	0.000	100
Gc	12 Jul	13.245	22.075	8.830	0.000	4.415	8.830	2.208	2.208	0.000	0.000	6.623	6.623	0.000	0.000	17.660	0.000	0.000	0.000	0.000	0.000	5.298	1.987	0.000	0.000	100
Gc	12 Jul	2.955	4.298	9.401	0.000	1.612	4.835	1.343	1.343	0.000	3.223	2.417	4.835	1.880	0.000	59.898	0.000	0.000	0.000	0.081	0.000	0.806	1.074	0.000	0.000	100
Gc	12 Jul	13.245	22.075	8.830	0.000	4.415	8.830	2.208	2.208	0.000	0.000	6.623	6.623	0.000	0.000	17.660	0.000	0.000	0.000	0.000	0.000	5.298	1.987	0.000	0.000	100
Gc	27 Jul	0.000	27.076	0.000	0.000	4.513	9.025	1.805	3.610	0.000	5.415	10.830	3.610	1.805	0.000	10.830	0.000	0.000	0.000	0.000	16.245	3.610	1.625	0.000	0.000	100
Gc	27 Jul	1.316	23.684	0.000	0.000	2.632	13.158	2.632	6.579	1.316	7.895	21.053	11.842	0.000	0.000	3.947	0.000	0.000	0.000	0.000	2.632	0.000	1.316	0.000	0.000	100
Gc	27 Jul	0.000	54.920	0.000	0.000	9.153	0.000	0.000	9.153	0.000	0.000	0.000	0.000	13.730	0.000	0.000	1.144	0.000	0.000	0.000	0.000	4.577	0.458	0.000	6.865	100
Gc	27 Jul	0.000	40.336	0.000	6.723	0.000	10.084	6.723	10.084	0.000	0.000	8.403	11.765	0.000	0.000	0.000	0.000	0.000	0.000	0.000	2.017	3.361	0.504	0.000	0.000	100
Gc	27 Jul	0.000	0.000	37.736	0.000	0.000	0.000	0.000	9.434	0.000	0.000	9.434	18.868	0.000	0.000	0.000	0.000	0.000	9.434	0.000	5.660	9.434	0.000	0.000	0.000	100
Gc	12 Aug	0.000	21.583	0.000	0.000	0.000	0.000	3.597	3.597	0.000	0.000	0.000	0.000	0.000	0.000	28.777	0.000	1.799	35.971	0.000	0.000	0.000	1.079	0.000	3.597	100
Gc	12 Aug	0.000	29.557	14.778	12.315	0.000	0.000	0.000	12.315	0.000	7.389	4.926	0.000	6.158	0.000	0.000	2.217	2.217	6.158	0.000	0.000	0.000	0.739	0.000	1.232	100
Gc	12 Aug	1.127	54.397	5.637	5.637	0.000	2.255	2.255	3.382	0.000	1.127	2.255	2.255	2.818	0.000	0.000	0.000	2.537	2.255	0.000	2.255	9.019	0.789	0.000	0.000	100
Gc	12 Aug	0.789	40.237	5.917	4.734	11.834	2.367	0.000	6.312	0.000	10.651	3.945	3.945	0.000	0.000	0.000	0.000	1.381	3.156	0.000	0.631	0.789	0.947	0.000	2.367	100
Gc	12 Aug	2.107	29.505	9.484	0.000	8.430	5.269	0.000	8.430	2.213	5.269	2.845	3.161	3.161	0.000	0.000	0.000	3.161	5.269	0.000	5.479	4.215	0.948	0.000	1.054	100
Gc	12 Aug	0.000	40.332	3.559	0.000	10.676	0.000	0.000	11.862	0.000	3.915	4.745	5.931	0.000	1.186	0.000	0.000	1.186	2.966	0.000	1.779	9.490	1.186	0.000	1.186	100
Gc	12 Aug	0.000	53.004	0.000	0.000	10.601	0.000	0.000	14.134	0.000	3.534	3.534	7.067	0.000	0.000	0.000	0.000	0.000	0.000	0.000	0.000	0.000	1.060	0.000	7.067	100
Gc	12 Aug	1.337	26.738	6.684	0.000	9.358	0.000	0.000	10.695	0.000	5.348	1.872	5.348	0.000	0.000	0.000	0.000	4.011	14.706	0.000	2.674	7.219	1.337	0.000	2.674	100
Gc	12 Aug	0.000	34.314	9.804	7.353	4.902	0.000	0.000	12.255	0.000	9.804	1.716	1.716	2.451	0.000	7.353	0.000	0.000	2.451	0.000	4.657	0.000	1.225	0.000	0.000	100
Gc	12 Aug	1.783	26.738	7.130	5.348	8.021	0.000	0.000	9.804	0.000	8.913	2.674	3.565	0.000	0.000	0.000	1.783	4.456	5.348	0.000	8.913	2.674	1.070	0.000	1.783	100
Gc	31 Aug	0.000	24.390	6.969	0.000	0.000	0.000	3.484	6.969	2.439	6.969	0.000	13.937	0.000	0.000	0.000	0.000	10.453	13.937	0.000	4.181	5.226	1.045	0.000	0.000	100
Gc	31 Aug	0.000	23.599	10.324	5.900	0.000	0.000	2.950	2.950	0.000	0.000	0.000	7.375	11.799	0.000	0.000	0.000	0.000	0.000	0.000	0.000	34.218	0.885	0.000	0.000	100
Gc	31 Aug	2.389	51.852	7.168	0.000	16.726	0.000	0.000	2.389	2.389	0.000	0.000	2.389	0.000	0.000	0.000	0.000	1.792	4.779	0.000	0.000	7.168	0.956	0.000	0.000	100
Gc	31 Aug	0.000	30.672	0.000	0.000	8.403	0.000	0.000	6.723	3.361	13.445	0.000	0.000	0.000	0.000	0.000	0.420	7.563	12.605	0.504	3.697	8.403	0.840	0.000	3.361	100
Gc	31 Aug	0.000	49.415	0.000	0.000	14.620	0.000	0.000	8.772	0.000	0.000	0.000	8.772	0.000	0.000	0.000	0.000	6.433	0.000	0.000	5.263	5.848	0.877	0.000	0.000	100
Gc	31 Aug	0.000	25.776	4.773	4.773	11.933	0.000	0.000	4.773	0.000	9.547	0.000	2.387	2.387	0.000	0.000	0.000	11.933	11.933	1.193	7.637	0.000	0.955	0.000	0.000	100
Pf	27 May	0.000	0.000	0.000	0.000	0.000	0.000	0.000	0.000	0.000	0.000	0.000	0.000	0.000	0.000	63.043	18.478	0.000	0.652	0.000	4.348	3.478	10.000	0.000	0.000	100
Pf	27 May	0.000	0.000	0.000	0.000	0.000	0.000	0.000	0.000	0.000	0.000	0.000	0.000	0.000	0.000	5.682	71.023	0.000	0.568	0.000	0.000	2.841	19.886	0.000	0.000	100
Pf	27 May	0.000	0.000	0.000	0.000	0.000	0.000	0.000	0.000	0.000	0.000	0.000	0.000	0.000	0.000	0.000	76.471	0.000	0.000	0.000	0.000	3.069	20.460	0.000	0.000	100
Pf	03 Jun	3.672	0.000	0.000	0.000	0.000	0.000	0.000	0.000	0.000	0.000	0.000	0.000	0.000	19.584	76.744	0.000	0.000	0.000	0.000	0.000	0.000	0.000	0.000	0.000	100
Pf	03 Jun	0.000	0.000	0.000	0.000	0.000	0.000	0.000	0.000	0.000	0.000	0.000	0.000	0.000	0.000	97.204	0.000	0.000	0.000	0.000	0.000	2.796	0.000	0.000	0.000	100
Pf	03 Jun	0.000	0.000	0.000	0.000	0.000	0.000	0.000	0.000	0.000	0.000	0.000	0.000	0.000	0.000	0.000	44.625	0.000	0.000	0.000	0.098	55.277	0.000	0.000	0.000	100
Pf	03 Jun	0.000	0.000	0.000	0.000	0.000	0.000	0.000	0.000	0.000	0.000	0.000	0.000	0.000	0.000	87.126	5.389	0.000	0.000	0.000	0.000	7.485	0.000	0.000	0.000	100
Pf	03 Jun	5.618	0.000	0.000	0.000	0.000	0.000	0.000	0.000	0.000	0.000	14.981	0.000	0.000	0.000	0.000	52.060	0.000	5.618	11.236	4.869	5.618	0.000	0.000	0.000	100
Pf	03 Jun	0.000	0.000	0.000	0.000	0.000	0.000	0.000	0.000	0.000	0.000	0.000	0.000	0.000	0.000	0.000	40.777	0.000	0.000	0.000	0.000	30.097	0.000	29.126	0.000	100
Pf	03 Jun	0.000	0.000	0.000	0.000	0.000	0.000	0.000	0.000	0.000	0.000	1.745	0.000	0.000	0.000	88.781	0.000	0.000	0.000	0.000	0.000	4.488	4.986	0.000	0.000	100
Pf	03 Jun	0.000	0.000	0.000	0.000	0.000	0.000	0.000	0.000	0.000	0.000	0.000	0.000	0.000	16.216	18.919	20.946	0.000	0.676	1.689	31.419	6.757	3.378	0.000	0.000	100
Pf	03 Jun	0.000	0.000	0.000	0.000	0.000	0.000	0.000	0.000	0.000	4.193	12.579	0.000	4.717	0.000	69.602	5.241	0.000	0.000	1.048	2.621	0.000	0.000	0.000	0.000	100
Pf	30 Jun	0.000	0.000	0.000	0.000	0.000	0.000	0.000	0.000	0.000	0.000	50.000	0.000	0.000	0.000	0.000	46.250	0.000	0.000	0.000	3.750	0.000	0.000	0.000	0.000	100
Pf	30 Jun	0.000	0.000	8.065	0.000	0.000	0.000	0.000	0.000	0.000	0.000	16.129	0.000	3.024	23.185	0.000	46.371	0.000	1.008	0.000	2.218	0.000	0.000	0.000	0.000	100
Pf	30 Jun	4.137	0.000	0.000	0.000	0.000	6.205	0.000	0.000	0.000	15.512	43.433	0.000	0.000	4.137	0.000	20.165	0.000	0.000	1.034	1.241	4.137	0.000	0.000	0.000	100
Pf	30 Jun	0.000	0.000	0.000	0.000	0.000	0.000	0.000	0.000	0.000	0.000	50.000	0.000	0.000	0.000	0.000	46.250	0.000	0.000	0.000	3.750	0.000	0.000	0.000	0.000	100
Pf	30 Jun	0.000	0.000	8.065	0.000	0.000	0.000	0.000	0.000	0.000	0.000	16.129	0.000	3.024	23.185	0.000	46.371	0.000	1.008	0.000	2.218	0.000	0.000	0.000	0.000	100
Pf	30 Jun	4.137	0.000	0.000	0.000	0.000	6.205	0.000	0.000	0.000	12.410	46.536	0.000	0.000	4.137	0.000	20.165	0.000	0.000	1.034	1.241	4.137	0.000	0.000	0.000	100
Pf	12 Jul	2.353	0.000	0.000	0.000	0.000	0.000	0.000	0.000	0.000	10.118	34.588	4.706	0.000	0.000	21.176	16.471	0.000	1.176	3.529	5.882	0.000	0.000	0.000	0.000	100
Pf	12 Jul	7.588	1.133	0.000	0.000	0.000	20.385	3.398	0.000	0.000	15.855	29.445	5.663	4.530	0.000	0.000	6.342	0.000	0.000	0.566	0.566	4.530	0.000	0.000	0.000	100
Pf	12 Jul	6.604	1.887	1.887	2.830	0.000	13.208	16.038	0.000	0.000	9.434	18.868	10.377	2.830	1.887	0.000	8.491	0.000	0.000	0.000	0.000	5.660	0.000	0.000	0.000	100
Pf	12 Jul	7.905	5.929	0.000	0.000	0.000	3.953	0.000	0.000	0.000	0.000	18.972	3.953	0.000	0.000	0.000	43.478	0.000	3.953	0.000	11.858	0.000	0.000	0.000	0.000	100
Pf	12 Jul	5.831	0.000	0.000	0.000	1.749	9.913	12.828	0.000	0.000	13.994	22.157	5.248	0.816	1.166	0.000	25.656	0.000	0.583	0.000	0.058	0.000	0.000	0.000	0.000	100
Pf	12 Jul	5.825	0.000	0.000	0.000	0.000	15.534	17.476	0.000	0.000	3.883	21.359	9.709	1.942	0.000	0.000	23.301	0.000	0.000	0.971	0.000	0.000	0.000	0.000	0.000	100
Pf	12 Jul	7.857	0.000	0.000	0.000	0.000	15.714	24.286	0.000	0.000	7.143	14.286	13.571	7.857	0.000	0.000	5.714	0.000	0.714	0.000	0.000	2.857	0.000	0.000	0.000	100
Pf	12 Jul	20.000	0.000	0.000	0.000	0.000	0.000	0.000	0.000	0.000	0.000	30.000	0.000	0.000	0.000	0.000	50.000	0.000	0.000	0.000	0.000	0.000	0.000	0.000	0.000	100
Pf	12 Jul	9.324	0.000	0.000	0.000	0.000	1.748	1.166	0.000	0.000	2.331	8.159	0.000	2.331	0.000	0.000	56.876	0.000	4.662	1.748	11.655	0.000	0.000	0.000	0.000	100
Pf	12 Jul	4.087	0.000	0.000	0.000	0.000	0.000	0.000	0.000	0.000	0.000	8.174	0.000	5.450	0.000	0.000	64.578	0.000	10.899	2.725	4.087	0.000	0.000	0.000	0.000	100
Pf	27 Jul	0.000	0.000	0.000	0.000	0.000	0.000	0.000	0.000	0.000	0.000	2.597	1.299	0.000	90.909	5.195	0.000	0.000	0.000	0.000	0.000	0.000	0.000	0.000	0.000	100
Pf	27 Jul	21.978	0.000	0.000	0.000	0.000	0.000	15.385	0.000	0.000	0.000	0.000	0.000	0.000	0.000	0.000	1.099	1.099	0.000	5.495	54.945	0.000	0.000	0.000	0.000	100
Pf	27 Jul	0.000	0.000	0.000	0.000	0.000	0.000	0.000	0.000	0.000	0.000	0.000	0.000	0.000	17.241	0.000	17.241	0.000	0.000	0.000	39.655	25.862	0.000	0.000	0.000	100
Pf	27 Jul	0.000	0.000	0.000	0.000	0.000	0.000	7.042	0.000	0.000	0.000	5.282	7.042	3.521	7.042	0.000	3.873	0.352	0.000	0.000	65.845	0.000	0.000	0.000	0.000	100
Pf	27 Jul	0.000	0.000	0.000	0.000	0.000	0.000	0.000	0.000	0.000	0.000	3.744	11.232	0.000	3.744	0.000	6.365	0.037	0.000	0.000	74.878	0.000	0.000	0.000	0.000	100
Pf	27 Jul	0.000	0.000	0.000	0.000	0.000	0.000	0.000	0.000	0.000	0.000	0.000	25.862	0.000	4.310	0.000	0.862	0.000	0.000	0.000	68.966	0.000	0.000	0.000	0.000	100
Pf	27 Jul	1.953	0.000	0.000	0.000	0.000	9.766	12.695	0.000	0.000	3.906	7.813	21.875	0.000	13.672	3.906	0.586	0.391	0.000	0.000	23.438	0.000	0.000	0.000	0.000	100
Pf	27 Jul	0.000	0.000	0.000	0.000	0.000	0.000	0.000	0.000	0.000	0.000	22.727	9.091	0.000	9.091	0.000	3.636	0.909	0.000	0.000	54.545	0.000	0.000	0.000	0.000	100
Pf	27 Jul	0.000	0.000	0.000	0.000	0.000	6.173	14.403	0.000	0.000	10.288	30.864	32.922	2.058	0.000	0.000	1.646	0.000	0.000	0.000	1.646	0.000	0.000	0.000	0.000	100
Pf	27 Jul	0.000	0.000	0.000	0.000	0.000	0.000	0.000	0.000	0.000	0.000	28.531	14.265	0.000	0.000	0.000	14.265	0.143	0.000	0.000	42.796	0.000	0.000	0.000	0.000	100
Pf	12 Aug	0.000	0.000	0.000	0.000	0.000	64.935	0.000	0.000	0.000	0.000	0.000	0.000	0.000	0.000	0.000	4.870	12.987	16.234	0.649	0.325	0.000	0.000	0.000	0.000	100
Pf	12 Aug	0.000	0.000	0.000	0.000	0.000	0.000	9.287	0.000	0.000	14.859	37.147	9.287	0.000	0.000	0.000	10.773	3.715	0.000	0.074	14.859	0.000	0.000	0.000	0.000	100
Pf	12 Aug	0.000	0.000	0.000	0.000	0.000	0.000	0.000	0.000	0.000	0.000	13.141	9.855	0.000	0.000	0.000	21.025	13.141	3.285	0.131	26.281	13.141	0.000	0.000	0.000	100
Pf	12 Aug	0.000	0.000	0.000	0.000	0.000	0.000	0.000	0.000	0.000	0.000	24.184	18.138	0.000	0.000	0.000	14.510	4.353	12.092	0.121	2.418	0.000	0.000	0.000	24.184	100
Pf	12 Aug	2.536	0.000	0.000	0.000	0.000	15.217	14.583	0.000	0.000	6.974	17.119	1.902	0.634	0.634	39.944	0.000	0.000	0.000	0.013	0.444	0.000	0.000	0.000	0.000	100
Pf	12 Aug	4.105	0.000	0.000	0.000	0.000	0.000	24.631	0.000	0.000	0.000	16.420	4.105	0.000	4.105	32.841	1.642	0.821	2.874	0.000	0.246	0.000	0.000	0.000	8.210	100
Pf	12 Aug	5.198	0.000	0.000	0.000	0.000	45.484	7.797	0.000	0.000	7.797	9.097	6.498	3.899	0.000	3.899	0.845	0.364	0.650	0.026	3.249	5.198	0.000	0.000	0.000	100
Pf	12 Aug	0.000	0.000	0.000	0.000	0.000	0.000	0.000	0.000	0.000	0.000	0.000	0.000	0.000	10.753	64.516	2.151	0.860	0.000	0.215	0.645	0.000	0.000	0.000	20.860	100
Pf	31 Aug	0.000	0.000	0.000	0.000	0.000	0.000	0.000	0.000	0.000	0.000	97.826	0.000	0.000	0.000	0.000	0.000	2.174	0.000	0.000	0.000	0.000	0.000	0.000	0.000	100
Pf	31 Aug	0.000	0.000	0.000	0.000	0.000	0.000	0.000	0.000	0.000	0.000	100.000	0.000	0.000	0.000	0.000	0.000	0.000	0.000	0.000	0.000	0.000	0.000	0.000	0.000	100
Pf	31 Aug	0.000	0.000	0.000	0.000	0.000	0.000	0.000	0.000	0.000	0.000	0.000	0.000	0.000	0.000	0.000	2.243	3.925	0.000	0.374	0.000	56.075	0.000	0.000	37.383	100
Pf	31 Aug	0.000	0.000	35.607	0.000	0.000	0.000	10.956	0.000	0.000	0.000	16.434	5.478	5.478	0.000	0.000	2.739	0.575	0.000	0.000	0.822	16.434	0.000	0.000	5.478	100
Pf	31 Aug	0.000	0.000	0.000	0.000	0.000	0.000	0.000	0.000	0.000	0.000	0.000	0.000	0.000	0.000	0.000	2.740	47.945	0.000	1.370	6.849	41.096	0.000	0.000	0.000	100
Pf	31 Aug	0.000	0.000	0.000	0.000	0.000	0.000	0.000	0.000	0.000	0.000	0.000	0.000	0.000	0.000	0.000	4.321	46.296	15.432	30.864	3.086	0.000	0.000	0.000	0.000	100
Pf	31 Aug	0.000	0.000	0.000	0.000	0.000	0.000	0.000	0.000	0.000	0.000	0.000	0.000	0.000	0.000	0.000	0.784	98.039	0.000	0.784	0.392	0.000	0.000	0.000	0.000	100
Pf	31 Aug	0.000	0.000	0.000	0.000	0.000	0.000	0.000	0.000	0.000	0.000	23.529	0.000	0.000	0.000	0.000	0.000	35.294	11.765	1.176	4.706	0.000	0.000	0.000	23.529	100
Pf	31 Aug	0.000	0.000	0.000	0.000	0.000	0.000	0.000	0.000	0.000	0.000	0.000	0.000	0.000	0.000	0.000	20.681	30.414	18.248	0.243	30.414	0.000	0.000	0.000	0.000	100
Ll	20 May	2.791	0.000	0.000	0.000	0.000	0.000	0.000	0.000	0.000	7.907	0.000	0.000	0.000	0.000	63.953	0.000	0.000	0.000	0.000	0.000	3.023	22.326	0.000	0.000	100
Ll	20 May	0.784	0.000	0.000	0.000	0.000	0.000	0.000	0.000	0.000	22.549	0.000	0.000	0.784	5.686	60.588	0.000	0.000	0.000	1.569	0.000	0.000	8.039	0.000	0.000	100
Ll	20 May	0.533	0.000	0.000	0.000	0.000	0.000	2.133	0.000	0.000	5.600	0.000	0.000	0.000	5.867	26.000	40.000	0.000	0.000	6.667	0.000	0.000	13.200	0.000	0.000	100
Ll	20 May	0.000	0.000	0.000	0.000	0.000	0.000	0.000	0.000	0.000	5.000	0.000	0.000	0.000	0.000	15.000	18.750	0.000	0.000	0.000	0.000	0.000	5.000	56.250	0.000	100
Ll	20 May	2.745	0.000	0.000	0.000	0.000	0.000	0.000	0.000	0.000	13.333	0.000	0.000	0.000	6.863	54.902	10.000	0.000	0.000	1.765	0.000	0.000	5.882	4.510	0.000	100
Ll	20 May	0.000	0.000	0.000	0.000	0.000	0.000	0.000	0.000	0.000	17.200	0.000	0.000	0.000	62.000	8.000	7.200	0.000	0.000	0.000	0.000	0.800	4.800	0.000	0.000	100
Ll	20 May	0.000	0.000	0.000	0.000	0.000	0.000	0.000	0.000	0.000	12.414	0.000	0.000	0.000	33.793	22.069	10.000	0.000	0.000	1.034	0.000	0.000	12.759	7.931	0.000	100
Ll	20 May	0.000	0.000	0.000	0.000	0.000	0.000	0.000	0.000	0.000	15.000	0.000	0.000	0.000	29.091	20.000	10.000	0.000	0.000	2.273	0.000	0.000	13.636	10.000	0.000	100
Ll	27 May	3.488	0.000	0.000	0.000	0.000	0.000	0.000	0.000	0.000	5.581	0.000	0.000	0.000	4.186	63.953	11.628	0.000	0.000	0.000	0.000	0.698	10.465	0.000	0.000	100
Ll	27 May	3.488	0.000	0.000	0.000	0.000	0.000	0.000	0.000	0.000	5.581	0.000	0.000	0.000	4.186	63.953	11.628	0.000	0.000	0.000	0.000	0.698	10.465	0.000	0.000	100
Ll	27 May	3.488	0.000	0.000	0.000	0.000	0.000	0.000	0.000	0.000	5.581	0.000	0.000	0.000	4.186	63.953	11.628	0.000	0.000	0.000	0.000	0.698	10.465	0.000	0.000	100
Ll	03 Jun	2.695	0.000	0.000	0.000	0.000	0.000	0.000	0.000	0.000	2.695	0.000	0.000	0.000	0.000	0.000	84.906	0.000	0.000	2.695	0.000	6.739	0.270	0.000	0.000	100
Ll	03 Jun	1.416	0.000	0.000	0.000	0.000	0.000	0.000	0.000	0.000	4.249	0.000	0.000	0.000	0.000	20.538	51.700	0.000	0.000	0.000	0.000	3.541	18.555	0.000	0.000	100
Ll	03 Jun	3.311	0.000	0.000	0.000	0.000	0.000	0.000	0.000	0.000	0.000	0.000	0.000	0.000	0.000	37.252	56.126	0.000	0.000	0.828	0.000	2.483	0.000	0.000	0.000	100
Ll	03 Jun	0.000	0.000	0.000	0.000	0.000	0.000	0.000	0.000	0.000	0.000	0.000	0.000	0.000	0.000	0.000	92.514	0.000	0.000	3.240	0.000	0.000	2.123	2.123	0.000	100
Ll	03 Jun	3.721	0.000	0.000	0.000	0.000	0.000	0.000	0.000	0.000	0.000	0.000	0.000	0.000	0.000	55.814	17.674	0.000	0.000	0.000	0.000	2.791	9.767	10.233	0.000	100
Ll	03 Jun	0.000	0.000	0.000	0.000	0.000	0.000	0.000	0.000	0.000	0.000	0.000	0.000	0.000	17.710	49.587	18.890	0.000	0.000	0.000	0.000	0.000	9.091	4.723	0.000	100
Ll	03 Jun	0.000	0.000	0.000	0.000	0.000	0.000	0.000	0.000	0.000	0.000	0.000	0.000	0.000	6.383	65.957	9.574	0.000	0.000	0.000	0.000	0.000	12.766	5.319	0.000	100
Ll	17 Jun	16.444	0.000	0.000	0.000	0.000	0.000	0.000	0.000	0.000	12.333	0.000	0.000	0.514	0.000	30.832	17.472	0.000	0.000	2.055	0.000	4.111	12.641	3.597	0.000	100
Ll	17 Jun	0.000	0.000	0.000	0.000	0.000	0.000	0.000	0.000	0.000	7.287	0.000	0.000	4.453	0.000	0.000	74.494	0.000	0.000	4.049	0.000	0.000	9.717	0.000	0.000	100
Ll	17 Jun	1.403	0.000	0.000	0.000	0.000	6.311	0.000	2.104	0.000	3.156	7.363	0.000	0.000	0.000	0.000	63.114	0.000	0.000	1.403	0.000	3.506	9.537	2.104	0.000	100
Ll	17 Jun	1.097	0.000	0.000	0.000	0.000	0.000	0.000	0.000	0.000	0.000	0.000	0.000	0.000	0.000	0.000	68.007	0.000	0.000	1.280	21.755	0.000	5.850	2.011	0.000	100
Ll	17 Jun	4.912	0.000	0.000	0.000	0.000	0.000	0.000	0.000	0.000	4.420	0.000	0.000	0.000	0.000	0.000	66.306	0.000	0.000	6.385	13.261	0.000	4.715	0.000	0.000	100
Ll	30 Jun	5.339	0.000	0.000	0.000	0.000	0.000	0.000	0.000	0.000	9.856	0.000	4.928	0.821	12.320	8.214	39.836	0.000	0.000	1.643	0.000	0.000	4.723	12.320	0.000	100
Ll	30 Jun	5.339	0.000	0.000	0.000	0.000	0.000	0.000	0.000	0.000	9.856	0.000	4.928	0.821	12.320	8.214	39.836	0.000	0.000	1.643	0.000	0.000	4.723	12.320	0.000	100
Ll	30 Jun	5.339	0.000	0.000	0.000	0.000	0.000	0.000	0.000	0.000	9.856	0.000	4.928	0.821	12.320	8.214	39.836	0.000	0.000	1.643	0.000	0.000	4.723	12.320	0.000	100
Ll	12 Jul	5.587	0.000	14.366	0.000	0.000	9.577	0.000	15.563	0.000	4.789	19.154	10.774	0.000	0.000	0.000	9.976	0.000	0.000	0.000	0.000	0.000	7.821	2.394	0.000	100
Ll	12 Jul	6.597	0.000	0.000	0.000	0.000	15.393	0.000	6.597	0.000	6.597	29.137	8.796	10.995	0.000	0.000	4.948	0.000	0.000	0.000	0.000	0.000	9.841	1.100	0.000	100
Ll	12 Jul	11.607	0.000	0.000	0.000	0.000	7.035	0.000	0.000	0.000	6.331	18.291	7.035	0.000	0.000	0.000	38.340	0.000	0.000	1.759	0.000	0.000	9.603	0.000	0.000	100
Ll	12 Jul	6.593	0.000	9.890	0.000	0.000	6.593	0.000	12.088	0.000	8.791	19.231	9.890	1.648	2.198	5.495	4.945	0.000	0.000	0.000	0.000	0.000	4.396	8.242	0.000	100
Ll	12 Jul	3.343	0.000	7.799	2.228	0.000	8.357	0.000	11.142	0.000	16.156	36.769	0.000	0.000	0.000	0.000	0.000	0.000	0.000	0.000	0.000	0.000	3.064	11.142	0.000	100
Ll	12 Jul	6.528	0.000	6.528	0.000	0.000	9.792	0.000	13.056	0.000	9.792	31.008	9.792	4.896	0.000	0.000	0.000	0.000	0.000	1.632	0.000	0.000	3.713	3.264	0.000	100
Ll	12 Jul	0.000	0.000	30.769	0.000	0.000	0.000	0.000	9.615	0.000	0.000	38.462	0.000	0.000	0.000	0.000	5.769	0.000	0.000	2.404	0.000	0.000	5.769	7.212	0.000	100
Ll	12 Jul	0.000	0.000	7.895	0.000	0.000	9.211	0.000	11.842	0.000	9.211	20.395	10.526	3.947	0.000	0.000	0.000	0.000	0.000	0.000	0.000	9.868	11.842	5.263	0.000	100
Ll	27 Jul	3.656	9.748	9.748	3.249	7.311	2.437	6.093	4.874	5.686	0.000	7.311	11.373	10.967	7.311	6.499	0.000	0.000	0.000	0.000	0.000	3.249	0.487	0.000	0.000	100
Ll	27 Jul	3.656	9.748	9.748	3.249	7.311	2.437	6.093	4.874	5.686	0.000	7.311	11.373	10.967	7.311	6.499	0.000	0.000	0.000	0.000	0.000	3.249	0.487	0.000	0.000	100
Ll	27 Jul	3.656	9.748	9.748	3.249	7.311	2.437	6.093	4.874	5.686	0.000	7.311	11.373	10.967	7.311	6.499	0.000	0.000	0.000	0.000	0.000	3.249	0.487	0.000	0.000	100
Rr	03 Jun	14.070	0.000	0.000	0.000	0.000	3.517	0.000	3.517	0.000	5.276	0.000	0.000	0.000	15.828	42.209	0.000	0.000	0.000	0.000	0.000	0.000	11.185	4.397	0.000	100
Rr	03 Jun	0.000	0.000	0.000	0.000	0.000	0.000	0.000	0.000	0.000	0.000	0.000	0.000	0.000	0.000	0.000	0.000	0.000	0.000	0.000	0.000	0.000	10.235	89.765	0.000	100
Rr	03 Jun	3.784	0.000	0.000	0.000	0.000	9.459	0.000	7.567	0.000	16.648	0.000	0.000	3.784	0.000	17.026	0.000	0.000	0.000	0.000	0.000	0.000	11.843	29.890	0.000	100
Rr	03 Jun	0.000	0.000	0.000	0.000	0.000	0.000	0.000	0.000	0.000	0.000	0.000	0.000	0.000	0.000	0.000	0.000	0.000	0.000	0.000	0.000	0.000	10.929	89.071	0.000	100
Rr	03 Jun	0.000	0.000	0.000	0.000	0.000	0.000	0.000	0.000	0.000	0.000	0.000	0.000	0.000	0.000	0.000	0.000	0.000	0.000	0.000	0.000	0.000	20.049	79.951	0.000	100
Rr	03 Jun	8.945	0.000	0.000	0.000	0.000	0.000	0.000	0.000	0.000	0.000	0.000	0.000	0.000	0.000	0.000	0.000	0.000	0.000	2.236	0.000	9.839	7.424	71.556	0.000	100
Rr	03 Jun	0.000	0.000	0.000	0.000	0.000	0.000	0.000	0.000	0.000	0.000	0.000	0.000	0.000	0.000	0.000	0.000	0.000	0.000	0.000	0.000	0.000	9.901	90.099	0.000	100
Rr	03 Jun	0.000	0.000	0.000	0.000	0.000	0.000	0.000	2.166	0.000	3.609	0.000	0.000	0.000	0.000	0.000	1.826	0.000	0.000	2.166	0.000	1.444	7.941	80.849	0.000	100
Rr	03 Jun	8.587	0.000	0.000	0.000	0.000	0.000	0.000	0.000	0.000	8.587	0.000	0.000	0.000	8.587	8.587	10.734	0.000	0.000	0.000	0.000	0.000	10.734	44.182	0.000	100
Rr	03 Jun	0.000	0.000	0.000	0.000	0.000	0.000	0.000	0.000	0.000	3.654	0.000	0.000	0.000	9.967	0.000	0.000	0.000	0.000	0.000	0.000	0.000	6.645	79.734	0.000	100
Rr	03 Jun	0.000	0.000	0.000	0.000	0.000	0.000	0.000	0.000	0.000	0.000	0.000	0.000	0.000	0.000	0.000	10.010	0.000	0.000	2.422	0.000	7.814	20.019	59.735	0.000	100
Rr	03 Jun	0.000	0.000	0.000	0.000	0.000	0.000	0.000	0.000	0.000	0.000	0.000	0.000	0.000	0.000	0.000	0.000	0.000	0.000	0.000	0.000	0.000	50.717	49.283	0.000	100
Rr	17 Jun	2.644	0.000	0.000	0.000	0.000	0.000	0.000	0.000	0.000	5.288	0.000	0.000	0.000	0.000	0.000	68.747	0.000	0.000	5.288	0.000	1.111	5.288	11.634	0.000	100
Rr	17 Jun	0.000	0.000	0.000	0.000	0.000	0.000	0.000	0.000	0.000	20.052	0.000	0.000	4.359	0.000	0.000	0.000	0.000	8.718	4.359	0.000	0.000	26.765	35.745	0.000	100
Rr	17 Jun	3.407	0.000	3.407	0.000	0.000	0.000	0.000	1.136	0.000	3.407	1.703	0.000	0.000	3.407	3.407	0.000	0.000	1.136	6.814	0.000	1.703	60.820	9.653	0.000	100
Rr	17 Jun	0.000	0.000	0.000	0.000	0.000	0.000	0.000	0.000	0.000	0.000	0.000	0.000	0.000	0.000	0.000	29.963	0.000	0.000	2.179	0.000	0.000	38.679	29.178	0.000	100
Rr	17 Jun	0.000	0.000	0.000	0.000	0.000	5.318	0.000	0.000	0.000	0.000	10.637	5.318	0.000	0.000	0.000	31.201	0.000	2.142	4.255	0.000	1.418	29.783	9.928	0.000	100
Rr	30 Jun	7.430	0.000	0.000	0.000	0.000	8.173	0.000	9.659	0.000	16.346	12.631	19.318	0.000	4.458	0.000	0.000	0.000	0.000	0.743	0.000	0.000	7.868	13.374	0.000	100
Rr	30 Jun	18.801	0.000	13.271	0.000	0.000	7.742	0.000	0.000	0.000	0.000	0.000	0.000	1.106	4.424	0.000	0.000	0.000	0.000	0.000	0.000	0.000	30.325	24.331	0.000	100
Rr	30 Jun	4.327	0.000	12.981	0.000	0.000	0.000	0.000	4.327	0.000	17.309	12.981	12.981	0.000	0.000	0.000	0.000	0.000	0.000	0.000	0.000	0.000	2.164	32.929	0.000	100
Rr	30 Jun	0.000	0.000	0.000	0.000	0.000	0.000	0.000	0.000	0.000	26.178	0.000	0.000	0.000	0.000	0.000	5.236	0.000	0.000	0.000	0.000	0.000	15.707	52.880	0.000	100
Rr	30 Jun	0.000	0.000	0.000	0.000	0.000	0.000	0.000	0.000	0.000	0.000	0.000	0.000	17.241	0.000	0.000	46.207	0.000	0.000	0.000	0.000	0.000	23.103	13.448	0.000	100
Rr	30 Jun	0.000	0.000	0.000	0.000	0.000	19.663	0.000	0.000	0.000	30.899	0.000	0.000	0.000	0.000	0.000	0.000	0.000	11.236	0.000	0.000	0.000	7.303	30.899	0.000	100
Rr	30 Jun	0.000	0.000	0.000	0.000	0.000	0.000	0.000	27.928	0.000	0.000	0.000	0.000	0.000	0.000	0.000	18.018	0.000	0.000	0.000	0.000	0.000	9.009	45.045	0.000	100
Rr	30 Jun	19.802	0.000	0.000	0.000	0.000	0.000	0.000	0.000	0.000	36.304	0.000	0.000	0.000	0.000	0.000	9.901	0.000	9.901	0.000	3.300	0.000	4.290	16.502	0.000	100
Rr	30 Jun	36.986	0.000	0.000	0.000	0.000	0.000	0.000	0.000	0.000	0.000	0.000	0.000	0.000	0.000	0.000	0.000	0.000	0.000	0.000	0.000	34.247	8.219	20.548	0.000	100
Rr	30 Jun	0.000	0.000	0.000	0.000	0.000	0.000	0.000	0.000	0.000	0.000	0.000	0.000	0.000	0.000	0.000	54.400	0.000	0.000	0.000	0.000	0.000	5.600	40.000	0.000	100
Rr	30 Jun	0.000	0.000	0.000	0.000	0.000	0.000	0.000	0.000	0.000	59.677	0.000	0.000	0.000	0.000	0.000	0.000	0.000	0.000	0.000	0.000	0.000	0.000	40.323	0.000	100
Rr	30 Jun	0.000	0.000	0.000	0.000	0.000	0.000	0.000	0.000	0.000	43.478	0.000	0.000	0.000	0.000	0.000	21.739	0.000	0.000	0.000	13.043	0.000	8.696	13.043	0.000	100
Rr	30 Jun	0.000	0.000	0.000	0.000	0.000	0.000	0.000	0.000	0.000	0.000	0.000	0.000	0.000	37.500	0.000	12.500	0.000	0.000	0.000	12.500	0.000	6.250	31.250	0.000	100
Rr	30 Jun	0.000	0.000	0.000	0.000	0.000	0.000	0.000	0.000	0.000	0.000	0.000	0.000	0.000	0.000	0.000	10.448	0.000	0.000	0.000	0.000	0.000	20.149	69.403	0.000	100
Rr	30 Jun	0.000	0.000	0.000	0.000	0.000	0.000	0.000	0.000	0.000	0.000	0.000	0.000	0.000	0.000	0.000	13.333	0.000	0.000	0.000	13.333	0.000	20.000	53.333	0.000	100
Rr	30 Jun	0.000	0.000	0.000	0.000	0.000	0.000	0.000	0.000	0.000	0.000	0.000	0.000	0.000	0.000	0.000	25.000	0.000	0.000	0.000	25.000	25.000	0.000	25.000	0.000	100
Rr	30 Jun	0.000	0.000	0.000	0.000	0.000	0.000	0.000	0.000	0.000	0.000	0.000	0.000	0.000	39.063	0.000	15.625	0.000	0.000	0.000	7.813	0.000	0.000	37.500	0.000	100
Rr	12 Jul	0.000	0.000	0.000	0.000	0.000	0.000	0.000	0.000	0.000	0.000	0.000	0.000	0.000	0.000	0.000	11.450	0.000	0.000	0.000	0.000	0.000	4.580	83.969	0.000	100
Rr	12 Jul	0.000	0.000	0.000	0.000	0.000	0.000	0.000	0.000	0.000	0.000	0.000	0.000	0.000	0.000	0.000	0.000	0.000	0.000	0.000	0.000	0.000	10.989	89.011	0.000	100
Rr	12 Jul	0.000	0.000	0.000	0.000	0.000	0.000	0.000	0.000	0.000	0.000	0.000	0.000	0.000	0.000	0.000	91.787	0.000	0.000	0.000	0.000	0.000	8.213	0.000	0.000	100
Rr	12 Jul	0.000	0.000	0.000	0.000	0.000	0.000	0.000	0.000	0.000	0.000	0.000	0.000	0.000	0.000	0.000	79.439	0.000	0.000	0.000	0.000	0.467	4.673	15.421	0.000	100
Rr	27 Jul	0.000	0.000	0.000	0.000	0.000	0.000	0.000	0.000	0.000	0.000	16.588	0.000	0.000	0.000	0.000	0.000	0.000	0.000	0.000	14.218	54.976	9.479	4.739	0.000	100
Rr	27 Jul	14.493	0.000	0.000	0.000	0.000	0.000	0.000	0.000	0.000	32.609	21.739	0.000	0.000	0.000	0.000	0.000	0.000	0.000	0.000	0.000	0.000	27.536	3.623	0.000	100
Rr	27 Jul	0.000	0.000	0.000	0.000	0.000	0.000	0.000	0.000	0.000	0.000	0.000	0.000	0.000	0.000	0.000	0.000	0.000	0.000	0.000	0.000	0.000	30.000	70.000	0.000	100
Rr	27 Jul	0.000	15.060	0.000	0.000	0.000	15.060	0.000	0.000	0.000	0.000	21.084	0.000	3.012	19.578	0.000	4.518	0.000	0.000	0.000	4.518	0.000	9.639	7.530	0.000	100
Rr	27 Jul	0.000	0.000	0.000	0.000	0.000	0.000	0.000	18.831	0.000	0.000	0.000	0.000	0.000	0.000	0.000	3.247	0.000	0.000	0.000	9.740	0.000	29.221	38.961	0.000	100
Rr	27 Jul	0.000	0.000	0.000	0.000	0.000	0.000	0.000	9.690	0.000	0.000	9.690	0.000	0.000	0.000	0.000	0.000	9.690	31.008	0.000	3.876	5.039	31.008	0.000	0.000	100
Rr	27 Jul	0.000	0.000	0.000	0.000	0.000	0.000	0.000	11.013	0.000	26.432	11.013	8.811	0.000	0.000	0.000	0.000	11.674	0.000	0.000	4.405	9.031	4.405	13.216	0.000	100
Rr	27 Jul	0.000	0.000	0.000	0.000	0.000	0.000	0.000	0.000	0.000	0.000	0.000	0.000	0.000	0.000	0.000	0.000	0.000	0.000	7.788	0.000	24.922	43.925	23.364	0.000	100
Rr	27 Jul	0.000	0.000	0.000	0.000	0.000	0.000	0.000	0.000	0.000	0.000	0.000	0.000	0.000	0.000	0.000	0.000	0.000	0.000	0.000	6.361	22.901	6.361	64.377	0.000	100
Rr	27 Jul	0.000	40.541	0.000	0.000	0.000	0.000	0.000	0.000	0.000	0.000	0.000	0.000	0.000	0.000	0.000	0.000	0.000	0.000	0.000	0.000	0.000	5.068	54.392	0.000	100
Rr	27 Jul	17.544	0.000	0.000	0.000	0.000	0.000	0.000	0.000	0.000	0.000	35.088	0.000	0.000	0.000	0.000	0.000	0.000	0.000	0.000	0.000	0.000	5.263	42.105	0.000	100

Rr	27 Jul	0.000	0.000	0.000	0.000	0.000	0.000	0.000	0.000	0.000	0.000	26.316	0.000	0.000	0.000	0.000	0.000	0.000	0.000	0.000	1.754	0.000	6.140	65.789	0.000	100
Rr	27 Jul	0.000	0.000	0.000	0.000	0.000	0.000	0.000	0.000	0.000	0.000	26.718	13.359	0.000	11.450	0.000	0.000	0.000	0.000	0.000	0.000	11.450	17.939	19.084	0.000	100
Rr	27 Jul	0.000	0.000	0.000	0.000	0.000	0.000	0.000	0.000	0.000	0.000	0.000	0.000	0.000	0.000	0.000	0.000	0.000	0.000	0.000	0.000	0.000	32.515	67.485	0.000	100
Rr	27 Jul	0.000	0.000	0.000	0.000	0.000	0.000	0.000	0.000	0.000	0.000	0.000	0.000	0.000	0.000	0.000	0.000	0.000	0.000	8.140	0.000	91.860	0.000	0.000	0.000	100
Rr	27 Jul	2.789	0.000	0.000	0.000	0.000	0.000	0.000	15.342	0.000	18.131	25.105	15.342	0.000	0.000	0.000	0.000	0.000	0.000	0.000	0.000	0.000	5.160	18.131	0.000	100
Rr	12 Aug	0.000	0.000	0.000	0.000	0.000	0.000	0.000	0.000	0.000	0.000	0.000	0.000	0.000	0.000	79.887	0.000	0.000	0.000	0.000	0.000	0.000	9.962	10.150	0.000	100
Rr	12 Aug	6.935	0.000	0.000	0.000	0.000	0.000	0.000	0.000	0.000	27.739	27.739	0.000	0.000	0.000	0.000	0.000	31.207	0.000	0.000	0.000	2.774	3.606	0.000	0.000	100
Rr	12 Aug	0.000	0.000	0.000	0.000	0.000	0.000	0.000	28.090	0.000	0.000	0.000	0.000	11.236	0.000	0.000	0.000	10.112	0.000	0.000	0.000	0.000	24.719	25.843	0.000	100
Rr	12 Aug	0.000	0.000	0.000	0.000	0.000	0.000	0.000	0.000	0.000	9.940	0.000	17.893	0.000	17.893	24.453	0.000	0.000	0.000	0.000	0.000	0.000	12.922	16.899	0.000	100
Rr	12 Aug	0.000	0.000	0.000	0.000	0.000	0.000	0.000	0.000	0.000	5.538	0.000	0.000	0.000	0.000	0.000	2.373	11.867	23.734	4.747	0.000	13.054	14.636	24.051	0.000	100
Rr	12 Aug	5.650	11.299	0.000	0.000	0.000	0.000	0.000	0.000	0.000	0.000	0.000	0.000	0.000	0.000	0.000	5.650	14.548	21.186	4.944	0.000	0.000	7.062	29.661	0.000	100
Rr	12 Aug	0.000	0.000	0.000	0.000	0.000	0.000	0.000	0.000	0.000	0.000	0.000	0.000	0.000	0.000	0.000	0.000	54.683	15.106	15.106	0.000	0.000	0.000	15.106	0.000	100
Rr	12 Aug	0.000	0.000	0.000	0.000	0.000	4.864	0.000	9.728	0.000	0.000	0.000	0.000	1.946	0.000	0.000	9.728	25.000	7.782	4.864	0.000	0.097	1.946	34.047	0.000	100
Rr	12 Aug	4.520	0.000	0.000	0.000	0.000	0.000	0.000	0.000	0.000	0.000	0.000	13.559	0.000	0.000	0.000	0.000	29.379	9.040	11.299	0.000	0.000	9.605	22.599	0.000	100
Rr	12 Aug	0.000	0.000	0.000	14.218	0.000	0.000	0.000	13.033	0.000	0.000	0.000	0.000	2.370	0.000	14.692	0.000	21.327	15.403	5.924	0.000	0.000	4.739	8.294	0.000	100
Rr	31 Aug	0.000	0.000	0.000	0.000	0.000	0.000	0.000	0.000	0.000	0.000	0.000	0.000	0.000	0.000	0.000	0.000	42.748	30.534	7.634	0.000	0.000	19.084	0.000	0.000	100
Rr	31 Aug	0.000	0.000	0.000	0.000	0.000	0.000	0.000	0.000	0.000	0.000	0.000	0.000	0.000	0.000	0.000	0.000	52.885	10.577	20.192	0.000	0.000	5.769	10.577	0.000	100
Rr	31 Aug	0.000	0.000	0.000	0.000	0.000	0.000	0.000	0.000	0.000	0.000	0.000	0.000	0.000	0.000	0.000	5.660	72.569	0.000	14.514	0.000	0.000	7.257	0.000	0.000	100
Rr	31 Aug	0.000	0.000	0.000	0.000	0.000	0.000	0.000	0.000	0.000	0.000	0.000	0.000	0.000	0.000	0.000	0.000	42.810	16.340	24.510	0.000	0.000	0.000	16.340	0.000	100
Rr	31 Aug	0.000	0.000	0.000	0.000	0.000	0.000	0.000	13.072	0.000	17.429	0.000	0.000	0.000	0.000	0.000	0.000	28.322	0.000	19.608	0.000	0.000	8.497	13.072	0.000	100
Rr	31 Aug	9.091	0.000	0.000	0.000	0.000	0.000	0.000	0.000	0.000	0.000	0.000	0.000	0.000	0.000	0.000	0.000	54.545	0.000	15.152	0.000	0.000	7.576	13.636	0.000	100
Rr	31 Aug	0.000	0.000	0.000	0.000	0.000	0.000	0.000	0.000	0.000	0.000	0.000	0.000	0.000	0.000	0.000	0.000	56.897	21.552	0.000	0.000	0.000	10.776	10.776	0.000	100
Rr	31 Aug	0.000	0.000	0.000	0.000	0.000	0.000	0.000	0.000	0.000	0.000	0.000	0.000	0.000	0.000	0.000	0.000	56.679	0.000	21.059	0.000	0.000	6.619	15.644	0.000	100
Rr	31 Aug	0.000	0.000	0.000	0.000	0.000	0.000	0.000	0.000	0.000	0.000	0.000	0.000	0.000	0.000	0.000	0.000	39.850	0.000	0.000	0.000	0.000	30.075	30.075	0.000	100
Rr	31 Aug	0.000	0.000	0.000	0.000	0.000	0.000	0.000	0.000	0.000	0.000	0.000	0.000	0.000	0.000	0.000	0.000	25.953	9.534	11.547	0.000	3.178	8.475	41.314	0.000	100
Rr	31 Aug	0.000	0.000	0.000	0.000	0.000	0.000	0.000	0.000	0.000	0.000	0.000	0.000	0.000	0.000	0.000	0.000	19.672	0.000	37.705	0.000	0.000	9.836	32.787	0.000	100
Rr	31 Aug	0.000	0.000	0.000	0.000	0.000	0.000	0.000	0.000	0.000	0.000	0.000	0.000	0.000	0.000	0.000	1.106	43.584	0.000	5.531	0.000	0.000	29.867	19.912	0.000	100
Ga	20 May	1.342	0.000	0.000	0.000	0.000	0.000	0.000	0.000	0.000	2.685	0.000	0.000	0.000	14.094	0.000	79.960	0.000	0.000	0.000	0.537	0.000	1.383	0.000	0.000	100
Ga	20 May	2.222	0.000	0.000	0.000	0.000	0.000	0.000	0.000	0.000	27.778	0.000	0.000	3.889	0.000	0.000	63.633	0.000	0.000	0.000	1.778	0.000	0.700	0.000	0.000	100
Ga	20 May	0.000	0.000	0.000	0.000	0.000	2.120	0.000	0.000	0.000	4.594	0.000	0.000	0.000	0.000	0.000	87.286	0.000	0.000	0.000	0.565	4.841	0.594	0.000	0.000	100
Ga	20 May	0.000	0.000	0.000	0.000	0.000	0.000	0.000	0.000	0.000	0.000	0.000	0.000	3.385	0.000	0.000	87.754	0.000	0.000	0.000	0.000	7.692	1.169	0.000	0.000	100
Ga	20 May	1.572	0.000	0.000	0.000	0.000	0.000	0.000	0.000	0.000	9.277	0.000	0.000	3.538	0.000	0.000	82.634	0.000	0.000	0.000	2.516	0.000	0.464	0.000	0.000	100
Ga	20 May	2.326	0.000	0.000	0.000	0.000	0.000	0.000	0.000	0.000	21.395	0.000	0.000	0.000	0.000	0.000	75.953	0.000	0.000	0.000	0.000	0.000	0.326	0.000	0.000	100
Ga	20 May	17.021	0.000	0.000	0.000	0.000	0.000	0.000	0.000	0.000	5.106	0.000	0.000	0.000	0.000	0.000	54.809	0.000	0.000	0.000	1.362	21.277	0.426	0.000	0.000	100
Ga	20 May	8.182	0.000	0.000	0.000	0.000	0.000	0.000	0.000	0.000	30.273	0.000	0.000	4.545	26.818	0.000	29.691	0.000	0.000	0.000	0.000	0.000	0.491	0.000	0.000	100
Ga	20 May	5.280	0.000	0.000	0.000	0.000	0.000	0.000	0.000	0.000	3.840	0.000	0.000	0.000	0.000	0.000	89.976	0.000	0.000	0.000	0.320	0.000	0.584	0.000	0.000	100
Ga	20 May	0.000	0.000	0.000	0.000	0.000	0.000	0.000	0.000	0.000	0.000	0.000	0.000	0.000	0.000	0.000	100.000	0.000	0.000	0.000	0.000	0.000	0.000	0.000	0.000	100
Ga	20 May	3.937	0.000	0.000	0.000	0.000	0.000	0.000	0.000	0.000	25.512	0.000	0.000	0.000	30.709	0.000	36.039	0.000	0.000	0.000	0.252	2.362	1.189	0.000	0.000	100
Ga	20 May	17.647	0.000	0.000	0.000	0.000	0.000	0.000	0.000	0.000	10.588	0.000	0.000	0.000	0.000	0.000	67.647	0.000	0.000	0.000	0.000	2.941	1.176	0.000	0.000	100
Ga	20 May	1.042	0.000	0.000	0.000	0.000	0.000	0.000	0.000	0.000	77.083	0.000	0.000	0.000	0.000	0.000	19.885	0.000	0.000	0.000	0.000	0.000	1.990	0.000	0.000	100
Ga	20 May	2.174	0.000	0.000	0.000	0.000	0.000	0.000	0.000	0.000	0.000	0.000	0.000	0.000	0.000	0.000	97.500	0.000	0.000	0.000	0.000	0.000	0.326	0.000	0.000	100
Ga	27 May	3.659	0.000	0.000	0.000	0.000	0.000	0.000	0.000	0.000	56.098	0.000	0.000	3.659	13.415	0.000	21.878	0.000	0.000	0.000	0.000	0.000	1.293	0.000	0.000	100
Ga	27 May	2.586	0.000	0.000	0.000	0.000	0.000	0.000	0.000	0.000	1.724	0.000	0.000	0.000	21.552	0.000	73.560	0.000	0.000	0.000	0.000	0.000	0.578	0.000	0.000	100
Ga	27 May	11.111	0.000	0.000	0.000	0.000	0.000	0.000	0.000	0.000	2.222	0.000	0.000	0.000	0.000	0.000	82.630	0.000	0.000	0.000	0.000	3.426	0.611	0.000	0.000	100
Ga	27 May	0.000	0.000	0.000	0.000	0.000	0.000	0.000	0.000	0.000	3.445	0.000	0.000	0.000	79.426	0.000	16.727	0.000	0.000	0.000	0.000	0.000	0.402	0.000	0.000	100
Ga	27 May	0.000	0.000	0.000	0.000	0.000	0.000	0.000	0.000	0.000	5.352	0.000	0.000	0.000	66.901	0.000	23.000	0.000	0.000	0.000	0.000	4.225	0.521	0.000	0.000	100
Ga	27 May	1.299	0.000	0.000	0.000	0.000	0.000	0.000	0.000	0.000	4.675	0.000	0.000	0.000	0.000	0.000	93.494	0.000	0.000	0.000	0.000	0.000	0.532	0.000	0.000	100
Ga	27 May	0.000	0.000	0.000	0.000	0.000	0.000	0.000	0.000	0.000	0.000	0.000	0.000	0.000	0.000	0.000	99.441	0.000	0.000	0.000	0.000	0.000	0.559	0.000	0.000	100
Ga	27 May	8.696	0.000	0.000	0.000	0.000	0.000	0.000	0.000	0.000	73.913	0.000	0.000	0.000	0.000	0.000	3.000	0.000	0.000	0.000	0.000	13.043	1.348	0.000	0.000	100
Ga	27 May	0.000	0.000	0.000	0.000	0.000	0.000	0.000	0.000	0.000	0.000	0.000	0.000	0.000	0.000	0.000	97.963	0.000	0.000	0.000	0.000	0.000	2.037	0.000	0.000	100
Ga	27 May	5.714	0.000	0.000	0.000	0.000	0.000	0.000	0.000	0.000	6.857	0.000	0.000	0.000	8.571	0.000	78.200	0.000	0.000	0.000	0.000	0.000	0.657	0.000	0.000	100
Ga	03 Jun	0.509	0.000	0.000	0.000	0.000	0.000	0.000	0.000	0.000	6.065	0.000	0.000	0.000	92.593	0.000	0.000	0.000	0.000	0.000	0.000	0.000	0.833	0.000	0.000	100
Ga	03 Jun	0.000	0.000	0.000	0.000	0.000	0.000	0.000	0.000	0.000	0.000	0.000	0.000	0.000	0.000	0.000	3.026	0.000	0.000	0.000	0.000	96.316	0.658	0.000	0.000	100
Ga	03 Jun	1.205	0.000	0.000	0.000	0.000	0.000	0.000	0.000	0.000	7.831	0.000	0.000	0.000	85.542	0.000	0.000	0.000	0.000	0.000	0.000	4.819	0.602	0.000	0.000	100
Ga	03 Jun	10.200	0.000	0.000	0.000	0.000	0.000	0.000	0.000	0.000	55.000	0.000	0.000	0.000	20.000	0.000	2.300	0.000	0.000	0.000	0.000	11.000	1.500	0.000	0.000	100
Ga	03 Jun	1.897	0.000	0.000	0.000	0.000	0.000	0.000	0.000	0.000	62.069	0.000	0.000	0.000	6.897	0.000	23.793	0.000	0.000	0.000	3.862	0.000	1.483	0.000	0.000	100
Ga	03 Jun	1.047	1.744	0.000	0.000	0.000	0.000	0.000	0.000	0.000	11.628	0.000	0.000	0.000	79.070	0.000	0.401	0.000	0.000	0.000	0.000	5.233	0.878	0.000	0.000	100
Ga	27 Jul	4.545	0.000	0.000	11.364	0.000	0.000	0.000	29.545	0.000	0.000	9.091	8.182	32.273	0.000	0.000	0.000	0.000	0.000	0.000	0.000	5.000	0.000	0.000	0.000	100
Ga	27 Jul	6.944	22.222	0.000	0.000	0.000	6.944	0.000	6.250	0.000	0.000	0.000	0.000	47.917	0.000	0.000	0.000	0.000	0.000	0.000	0.000	6.944	2.278	0.500	0.000	100
Ga	27 Jul	0.000	0.000	0.000	0.000	0.000	0.000	0.000	7.317	0.000	0.000	0.000	0.000	63.415	19.512	0.000	0.000	0.000	0.000	0.000	0.000	9.756	0.000	0.000	0.000	100
